# Collagen Binding Proteins of Gram-Positive Pathogens

**DOI:** 10.3389/fmicb.2021.628798

**Published:** 2021-02-05

**Authors:** Srishtee Arora, Jay Gordon, Magnus Hook

**Affiliations:** Center for Infectious and Inflammatory Diseases, Institute of Biosciences and Technology, Texas A&M Health Science Center, Houston, TX, United States

**Keywords:** Gram-positive bacteria, collagen binding proteins, collagen-like proteins, surface proteins, collagen

## Abstract

Collagens are the primary structural components of mammalian extracellular matrices. In addition, collagens regulate tissue development, regeneration and host defense through interaction with specific cellular receptors. Their unique triple helix structure, which requires a glycine residue every third amino acid, is the defining structural feature of collagens. There are 28 genetically distinct collagens in humans. In addition, several other unrelated human proteins contain a collagen domain. Gram-positive bacteria of the genera *Staphylococcus*, *Streptococcus*, *Enterococcus*, and *Bacillus* express cell surface proteins that bind to collagen. These proteins of Gram-positive pathogens are modular proteins that can be classified into different structural families. This review will focus on the different structural families of collagen binding proteins of Gram-positive pathogen. We will describe how these proteins interact with the triple helix in collagens and other host proteins containing a collagenous domain and discuss how these interactions can contribute to the pathogenic processes.

## Introduction

Collagen is the most abundant protein in the human body and an integral component of the extracellular matrix (ECM) (Shoulders and Raines, 2009). The ECM is a complex proteinaceous network that provides structural support to tissues along with the necessary signaling for cell adhesion, migration, and growth as well as for tissue development and regeneration (Frantz et al., 2010). Collagen plays a critical role in the functional integrity of most tissues including bone, skin, tendon, and cartilage (Burgeson and Nimni, 1992; Frantz et al., 2010). Collagen can also be the target of surface-anchored adhesins and other virulence factors produced by both Gram-positive and Gram-negative pathogens (Harrington, 1996; Singh et al., 2012; Zhang et al., 2015; Duarte et al., 2016; Paulsson and Riesbeck, 2018; Vaca et al., 2020). Of these, the cell wall anchored collagen binding proteins in Gram-positive bacteria have been more extensively studied, and will be reviewed here.

There are 28 identified types of collagens in humans (Table 1; Ricard-Blum, 2011). Each collagen molecule is formed through the interactions of three protein polypeptides known as α-strands. The α-strands come together to form a canonical right-handed triple helical structure termed the triple helix domain (Kadler et al., 2007; Ricard-Blum, 2011). Triple helices can be formed by association of identical α-strands to form a homotrimer or be composed of different α-strands (heterotrimer) (Ricard-Blum, 2011). The triple helix domain is a flexible rod-shaped structure held together through inter-chain hydrogen bonding (Kadler et al., 2007; Shoulders and Raines, 2009; Ricard-Blum, 2011). The triple helix is defined by Gly-X-X’ amino acid repeats with X and X’ commonly representing proline and 4-hydroxyproline, respectively (Shoulders and Raines, 2009). Glycine residues are required every 3rd residue as any other residue would result in steric hindrance and helix destabilization (Theocharis et al., 2016). Collagens also have non-triple helical domains at their N- and C-termini, which are referred here as “non-collagenous” domains. In addition to the conventional collagens, several other mammalian proteins contain collagenous domains (Fraser and Tenner, 2008; Zani et al., 2015; PrabhuDas et al., 2017; Casals et al., 2019).

**TABLE 1:** The collagen family.

**Collagen type**	**Classification**	**Chain composition**	**Tissue distribution**	**Function**	**References**
I	Fibril-forming	α1[I]_2_α2[I] α1[I]_3_	Abundant and present in most connective tissues and interstitial membranes	Key structural component	Ricard-Blum, 2011; Henriksen and Karsdal, 2016
II	Fibril-forming	α1[II]_3_	Cartilage, vitreous humor, intervertebral disk	Tissue integrity and resiliency to stress	Ricard-Blum, 2011; Gudmann and Karsdal, 2016
III	Fibril-forming	α1[III]_3_	Tissues containing type I collagen, especially embryonic skin and hollow organs like blood vessels, uterus and bowel	Structural component, wound healing, interacts with platelets in blood clotting cascade	Ricard-Blum, 2011; Nielsen and Karsdal, 2016a
IV	Network-forming	α1[IV]_2_ α2[IV] α3[IV] α4[IV] α5[IV] α5[IV]_2_ α6[IV]	Basement membranes	Barrier between tissue compartments, signaling	Ricard-Blum, 2011; Sand et al., 2016
V	Fibril-forming	α1[V]_3_ α1[V]_2_ α2[V] α1[V] α2[V] α3[V]	Tissues containing type I collagen	Regulates collagen fibrillogenesis	Ricard-Blum, 2011; Leeming and Karsdal, 2016
VI	Beaded-filament-forming	α1[VI] α2[VI] α3[VI] α1[VI] α2[VI] α4[VI]	Most connective tissues	Modulates stiffness and mechanical properties of extracellular matrix, signaling	Ricard-Blum, 2011; Sun and Karsdal, 2016a
VII	Anchoring fibrils	α1[VII]_2_ α2[VII] α1[VII]_3_	Many tissues	Stability of extracellular matrix	Ricard-Blum, 2011; Mortensen and Karsdal, 2016
VIII	Network-forming	α1[VIII]_3_ α2[VIII]_3_ α1[VIII]_2_ α2[VIII] α1[VIII] α2[VIII]_2_	Descemet’s membrane, heart, brain, liver, lung, muscles and around chondrocytes in cartilage	Structural component, signaling	Ricard-Blum, 2011; Hansen and Karsdal, 2016
IX	FACIT	α1[IX] α2[IX] α3[IX]	Tissues containing type II collagen	Stabilization of the fibrillar collagen network, limits collagen fibril diameter	Ricard-Blum, 2011; He and Karsdal, 2016
X	Network-forming	α1[X]_3_	Hypertrophic cartilage	Endochondral ossification	Shen, 2005; Ricard-Blum, 2011
XI	Fibril-forming	α1[XI] α2[XI] α3[XI] α1[XI] α1[V] α3[XI]	Tissues containing type II collagen	Regulate fibrillogenesis of type II collagen fibrils, nucleator for collagen types I and II fibrillogenesis	Ricard-Blum, 2011; Luo and Karsdal, 2016
XII	FACIT	α1[XII]_3_	Tissues containing type I collagen	Osteoblast/osteocyte differentiation, skin homeostasis and repair, tendon development, regulation of fibrillogenesis	Ricard-Blum, 2011; Izu et al., 2020; Schönborn et al., 2020
XIII	MACIT	α1[XIII]_3_	Many tissues but present in low amounts	Plays a role in bone formation, presynaptic and postsynaptic maturation and integrity	Ricard-Blum, 2011; Zainul et al., 2018
XIV	FACIT	α1[XIV]_3_	Tissues containing type I collagen	Regulates fibrillogenesis by limiting fibril diameter	Ricard-Blum, 2011; Manon-Jensen and Karsdal, 2016
XV	Multiplexin	α1[XV]_3_	Basement membrane	Crosslinks collagen type I and III fibrils	Ricard-Blum, 2011; Arvanitidis and Karsdal, 2016
XVI	FACIT	α1[XVI]_3_	Many tissues	Stability of extracellular matrix, signaling	Ricard-Blum, 2011; Sand and Karsdal, 2016
XVII	MACIT	α1[XVII]_3_	Hemidesmosomes	Adhesion of epithelial cells to extracellular matrix, teeth formation	Ricard-Blum, 2011; Sun and Karsdal, 2016b
XVIII	Multiplexin	α1[XVIII]_3_	Basement membrane	Integrity of basement membrane, inhibit angiogenesis and tumor growth	Ricard-Blum, 2011; Bager and Karsdal, 2016
XIX	FACIT	α1[XIX]_3_	Basement membrane	Acts as a cross-bridge between collagen fibrils and other extracellular molecules	Ricard-Blum, 2011; Nielsen and Karsdal, 2016b
XX	FACIT	α1[XX]_3_	Cornea, minor component of multiple connective tissues	Specific role unknown	Ricard-Blum, 2011; Willumsen and Karsdal, 2016
XXI	FACIT	α1[XXI]_3_	Many tissues	Acts as a cross-bridge between collagen fibrils and other extracellular matrix molecules	Ricard-Blum, 2011; Kehlet and Karsdal, 2016
XXII	FACIT	α1[XXII]_3_	Tissue junction in skeletal and heart muscle	Plays a role in vascular stability	Ricard-Blum, 2011; Ton et al., 2018
XXIII	MACIT	α1[XXIII]_3_	Cornea, lung, cartilage, amnion	Induce keratinocyte adhesion and spreading, cancer cell metastasis	Ricard-Blum, 2011; Veit et al., 2011; Spivey et al., 2012
XXIV	Fibril-forming	α1[XXIV]_3_	Bone, cornea	Regulation of osteoblast differentiation and mineralization	Ricard-Blum, 2011; Wang et al., 2012
XXV	MACIT	α1[XXV]_3_	Brain, neurons	Fusion of myoblasts into myofibers, regulates intramuscular motor innervation	Ricard-Blum, 2011; Tanaka et al., 2014; Gonçalves et al., 2019
XXVI	–	α1[XXVI]_3_	Testis, ovary	Development of reproductive tissues	Sato et al., 2002; Ricard-Blum, 2011
XXVII	Fibril-forming	α1[XXVII]_3_	Cartilage, eye, ear, lung, colon	Structural role in the pericellular extracellular matrix, transition of cartilage to bone	Ricard-Blum, 2011; Luo et al., 2017
XXVIII	–	α1[XXVIII]_3_	Dorsal root ganglia, peripheral nerves, in low amounts in skin and calvaria	Specific role unknown	Ricard-Blum, 2011; Gebauer et al., 2016

Bacterial surface proteins contribute to pathogenic processes and play a critical role in mediating adhesion to host cells and tissues, enabling colonization, invasion, and biofilm formation (Foster et al., 2014; Foster, 2019). In addition, binding of bacterial surface proteins to host ligands can lead to evasion of the host defense systems (Foster et al., 2014; Foster, 2019). In Gram-positive bacteria, different classes of surface proteins exist: (1) lipoproteins, (2) proteins covalently anchored to the cell wall, (3) pilus proteins, (4) non-covalently surface-associated proteins, and (5) transmembrane proteins (Desvaux et al., 2006; Fischetti, 2019). Lipoproteins are proteins covalently attached to membrane lipids via their N-terminus (Desvaux et al., 2006). Cell wall anchored proteins and pilus proteins are anchored to the cell wall by the action of enzymes called sortases (Desvaux et al., 2006). Sortases mediate covalent linking of proteins to the peptidoglycan through a transpeptidase reaction, and can also enable assembly of surface pilus and anchor the pilus onto the peptidoglycan layer (Ton-That and Schneewind, 2004; Desvaux et al., 2006; Fischetti, 2019). Lastly, non-covalently surface associated proteins contain cell wall binding domains (Desvaux et al., 2006; Fischetti, 2019).

Bacterial surface proteins are modular multi-domain proteins that can often be grouped into structural families based on their structural similarities. Multiple structurally related families of proteins have been identified in the literature (Waldemarsson et al., 2006; Foster et al., 2014; Frost et al., 2017; Foster, 2019; Taglialegna et al., 2020). Notables examples of structural families in Gram-positive bacteria include the MSCRAMMs (microbial surface components recognizing adhesive matrix molecules) (Foster et al., 2014; Foster, 2019), serine-rich repeat proteins (Lizcano et al., 2012), and M-proteins (Fischetti, 2016). In this review, we will describe collagen binding proteins present on the surface of Gram-positive pathogens that are human pathogens. This review will focus on structural families where more than one protein with structural similarity has been reported to bind collagen directly. Some proteins reported in the literature use fibronectin as a bridging molecule to bind collagen, e.g., streptococcal fibronectin binding protein 1 (SfbI) of *Streptococcus pyogenes* (Dinkla et al., 2003a) and are not covered here.

## Types of Collagen

Collagens can be divided into different categories, which include fibrillar collagen, network forming collagen, FACITs (fibril-associated collagens with interrupted triple helices), MACITs (membrane-associated collagens with interrupted triple helices), anchoring fibrils, beaded-filament-forming collagens, and MULTIPLEXIN (multiple triple-helix domains and interruptions). These major classes of collagen will be discussed briefly below (Ricard-Blum, 2011; Theocharis et al., 2016). Collagen structure, chain composition, tissue distribution and functions are listed in Table 1.

Fibrillar collagen is the most common type of collagen in humans. Collagen types I, II, III, V, XI, XXIV, and XXVII all have a fibrillary configuration (Kadler et al., 2007; Theocharis et al., 2016). During fibrillogenesis, the protocollagen strands assemble into a triple helical formation called procollagen triple helix, which undergoes cleavage of N- and C-termini to generate tropocollagen triple helix molecule (Kadler et al., 2007; Shoulders and Raines, 2009; Theocharis et al., 2016). Tropocollagen triple helix molecule self-assembles into a D-staggered arrangement with a 67 nm periodicity to form collagen microfibrils [for further details on D-staggering, see Boudko and Bächinger (2016), Kadler (2017), Holmes et al. (2018)]. As a last step, collagen fibrils of diameter 15–500 nm are formed by crosslinking of collagen microfibrils (Kadler et al., 2007; Shoulders and Raines, 2009; Theocharis et al., 2016). Collagen fibrils in turn participate in forming larger structures such as ligaments and tendons (Ricard-Blum, 2011).

Network forming collagens include collagen types IV, VIII, and X, with collagen type IV being the archetype (Theocharis et al., 2016). Collagen type IV is found in the basement membrane along with other molecules such as laminin (Hohenester and Yurchenco, 2013; Theocharis et al., 2016). Unlike fibrillary collagens, the non-collagenous domains of these molecules are not cleaved and are utilized to form tail to tail interactions with other non-collagenous domains of collagen (Sundaramoorthy et al., 2002). Stabilizing tetramers are also formed via N-terminal head to head interactions. Once a mature network is formed, these collagens work to support the surrounding epithelial cell layer (Kadler et al., 2007).

Fibril-associated collagens with interrupted triple helices are relatively short flexible collagens that contain small triple helical regions interrupted by non-collagenous domains (Theocharis et al., 2016). Collagen types IX, XII, XIV, XVI, XIX, XX, XXI, and XXII have been reported as FACITs (Shoulders and Raines, 2009). Their primary role is to connect other collagen types together as well as with various ECM components (Theocharis et al., 2016). Collagen type IX, an archetypal FACIT, is covalently linked to collagen type II present in cartilage (Kadler et al., 2007; Ricard-Blum, 2011; Theocharis et al., 2016) and collagen type XIV binds to type I (Kadler et al., 2007; Ricard-Blum, 2011; Theocharis et al., 2016).

Membrane-associated collagens with interrupted triple helices are transmembrane proteins and contain a short N-terminal cytoplasmic tail, a transmembrane helix, and a collagenous C-terminal extracellular domain. These collagens can act as cellular receptors and facilitate cell adhesion and as soluble collagen in ECM upon cleavage (Ricard-Blum, 2011; Theocharis et al., 2016). Examples of MACITs include collagen types XIII, XXIII, and XXV and these are expressed by several cell types (Kadler et al., 2007; Theocharis et al., 2016).

Beaded filament collagens include collagen types VI, XXVI, and XXVIII with type VI being the most studied (Theocharis et al., 2016). Once these collagens are secreted from the cell, they arrange in an anti-parallel fashion to form dimers. Dimers then form tetramers through interactions with other dimers. Next, tetramers connect by their globular domains to form filaments where globular domains appear as beads (Kadler et al., 2007; Theocharis et al., 2016). Beaded filament collagens are found in various connective tissues, e.g., cartilage, bone, tendon, etc. (Fitzgerald et al., 2013).

Multiplexins include collagen types XV and XVIII and have not been studied extensively (Theocharis et al., 2016). They are localized to vascular and epithelial basement membranes and participate in bridging other collagens to underlying structures (Theocharis et al., 2016).

## Other Host Proteins With Collagen-Like Regions

G-X-X’ repeats are the defining feature of the collagen triple helix primary sequence. Proteins with collagen-like regions but not classified as conventional collagen have been identified in mammals and microbes (Pyagay et al., 2005; Tom Tang et al., 2005; Ricard-Blum, 2011; Yu et al., 2014; Casals et al., 2019). Mammalian proteins containing collagen-like domains include membrane proteins (e.g., scavenger receptors) (Ricard-Blum, 2011; Zani et al., 2015; PrabhuDas et al., 2017) and secreted proteins (e.g., human defense collagens) (Ricard-Blum, 2011; Casals et al., 2019).

Human defense collagens include members of the collectin family, ficolins, and C1q and TNF-related proteins (Casals et al., 2019). Members of the collectin family are surfactant protein A and D, mannan-binding lectin, collectin liver-1, collectin kidney-1, and the heterotrimeric Collectin CL-LK formed by the combination of collectin liver-1 and collectin kidney -1 (Casals et al., 2019). The ficolin family contains three ficolins: M-, H-, and L-ficolin (Casals et al., 2019). The C1q and TNF-related protein family only contains two members: C1q and adiponectin (Casals et al., 2019).

The defense collagens contain a N-terminal segment, a collagen-like region and a globular recognition domain that recognizes pathogen-associated molecular patterns and danger-associated molecular patterns (Fraser and Tenner, 2008; Casals et al., 2018, 2019). These proteins form multimeric structures and play an important role in pathogen clearance (Fraser and Tenner, 2008; Casals et al., 2018, 2019). The collagen-like regions of defense collagens vary in length and contain G-X-X’ repeats where X is often a proline, and X’ is often a hydroxylysine or a hydroxyproline (Casals et al., 2019). The collagen-like domains in human defense collagens serve two functions: (1) binding to associated proteases responsible for triggering the complement cascade and (2) binding cell receptors involved in clearance of pathogens and dead cells (Casals et al., 2019).

## Collagen Binding Proteins

### CNA-Like MSCRAMMs

In 1985 *Staphylococcus aureus* was reported to bind type I procollagen and soluble collagen type I (Carret et al., 1985; Holderbaum et al., 1985). Later the collagen “receptor” on *S. aureus* was identified as a 135 kDa cell wall-anchored protein and named Collagen Adhesin (CNA) (Speziale et al., 1986). Since then, bioinformatic analyses have identified homologous proteins in other Gram-positive bacteria. These include *Enterococcus faecalis* (Ace) (Rich et al., 1999), *Enterococcus faecium* (Acm) (Nallapareddy et al., 2003), *Streptococcus mutans* (Cnm) (Sato et al., 2004), *Streptococcus equi* (Cne) (Lannergard et al., 2003), *S. mutans* (Cbm) (Nomura et al., 2012), *Bacillus anthracis* (BA0871 and BA5258) (Xu et al., 2004a), *Erysipelothrix rhusiopathiae* (RspA and RspB) (Shimoji et al., 2003), and Acb from *Streptococcus gallolyticus* (Sillanpää et al., 2009; Table 2). Amongst these proteins, Cna and Ace are the best-studied members.

**TABLE 2:** Collagen binding CNA-like proteins.

**Protein name**	**Species**	**Collagen**	***K*_*D*_**	**References**
Cne	*S. equi*	I	125 nM	van Wieringen et al., 2010
		II	50 nM	van Wieringen et al., 2010
		III	100 nM	van Wieringen et al., 2010
Cbm	*S. mutans*	I	ND	Nomura et al., 2012, 2013
		III	ND	Nomura et al., 2013
		IV	ND	Nomura et al., 2013
Acm	*E. faecium*	I	3.8 μM	Nallapareddy et al., 2003
		IV	12.8 μM	Nallapareddy et al., 2003
Ace	*E. faecalis*	I	48 μM	Rich et al., 1999; Ross et al., 2012
		IV	ND	Nallapareddy et al., 2000
Cna	*S. aureus*	I	54 nM	Xu et al., 2004b; Ross et al., 2012
Acb	*S. gallolyticus*	I	45 nM	Sillanpää et al., 2009
		IV	0.3 μM	Sillanpää et al., 2009
		V	0.5 μM	Sillanpää et al., 2009
Cnm	*S. mutans*	I	ND	Sato et al., 2004; Nomura et al., 2013
		III	ND	Nomura et al., 2013
		IV	ND	Nomura et al., 2013
BA0871	*B. anthracis*	I	1.6–3.2 μM	Xu et al., 2004a
BA5258	*B. anthracis*	I	0.6–0.9 μM	Xu et al., 2004a

*ND stands for not determined.*

With the exception of Acb, all CNA-like proteins are anchored directly to the cell wall. Acb is unique and is a minor pilus protein of *S. gallolyticus* (Sillanpää et al., 2009) but has a predicted CNA-like structure. Furthermore, it shares 50–70% sequence identity with Acm, Cna, and Cne (Sillanpää et al., 2009).

#### Structure

**FIGURE 1:**
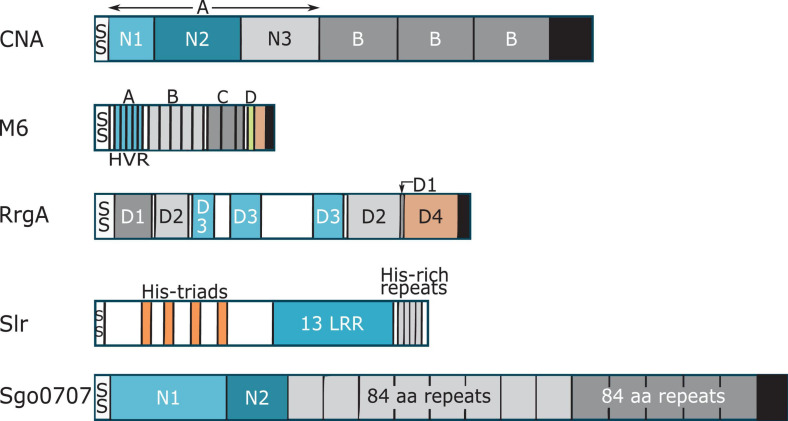
Cartoon representation showing domain organization of collagen binding proteins. Each protein has a signal peptide (SS) at the N-terminus. With the exception of Slr, each protein has a LPXTG motif, and a cell wall-spanning transmembrane region (black). Confirmed and putative collagen-binding domains in the proteins are colored in blue. Where two domains are involved in collagen binding, the first domain is light blue and the second domain is colored darker blue. Proline-glycine rich region of M6 protein is shown in salmon color.

**FIGURE 2:**
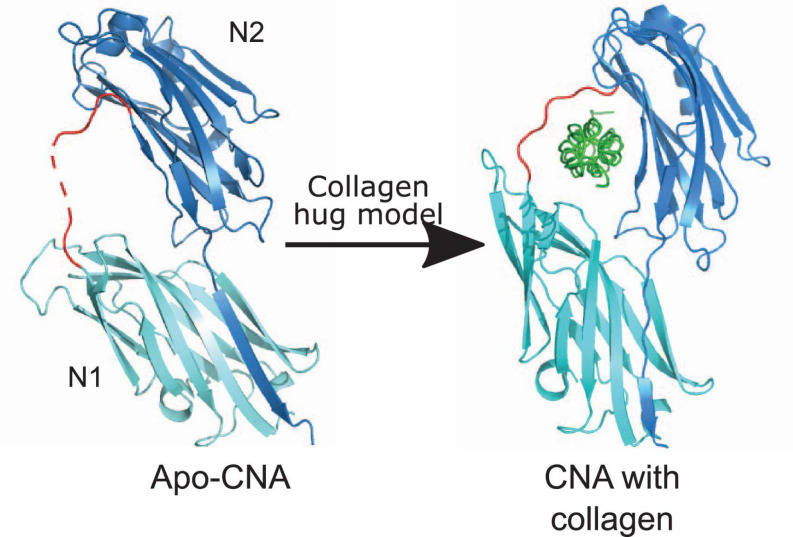
Collagen hug model. The N1N2 domains of CNA in both apo-form [PDB code: 2F68 (Zong et al., 2005)] and with collagen peptide [PDB code: 2F6A (Zong et al., 2005)] are shown. N2 and N1 domains are shown in dark blue and light teal color, respectively. CNA N1N2 domains are connected together by a linker shown in red. Collagen triple helix is shown in green. C-terminal extension of the N2 domain that forms a latch is shown as dark blue β-strand in N1 domain.

Collagen Adhesin is the prototype of Collagen-binding MSCRAMMs (Foster et al., 2014; Foster, 2019). CNA like proteins harbor a N-terminal signal sequence, an A-region, a variable number of characteristic B repeats, a C-terminal cell wall and membrane spanning region and a short cytoplasmic tail (Figure 1). The ligand-binding A-region of CNA-like proteins is further divided into two or three sub-domains: N1, N2, and N3 (Figure 1; Patti et al., 1993; Zong et al., 2005). X-ray crystallography of Cna and Ace N1N2 sub-domains revealed that these domains adopt IgG-like folds called Dev-IgG and are consequently composed of mostly β-sheets (Figure 2; Foster et al., 2014; Foster, 2019). The N1 and N2 domains are connected by a rather long (10 aa) hydrophobic linker region, which creates a hole of ∼15 Å between the two domains and provides flexibility in domain orientation (Figure 2; Zong et al., 2005). Additionally, proteins in the CNA-like MSCRAMM family have a variable number of B repeats depending upon the protein (Patti et al., 1994a; Kang et al., 2013). One B repeat is ∼180 aa long and is further divided into two ∼90 aa subdomains, D1 and D2. The D subdomains adopt an inverse IgG fold and together B repeats are thought to form a stalk projecting the ligand binding region away from the bacterial cell surface (Deivanayagam et al., 2000).

#### Binding Mechanism

The truncated N2 domain is the minimum collagen-binding region of CNA, although optimal binding is achieved by the N1N2 segments. The CNA N1N2 segment binds collagen type I with an affinity of 54 nM (Patti et al., 1993, 1995; Zong et al., 2005; Ross et al., 2012). Electron microscopy imaging of rCNA with collagen triple helix monomers revealed that CNA binds collagen at multiple sites, without any obvious preference for a “hot spot”. Surface plasmon resonance (SPR) studies of rCNA_31__–__344_ with synthetic collagen peptides further confirmed its preference for a triple helical structure (Zhang et al., 2015). CNA binds preferentially to cleaved collagen in damaged or inflamed tissues (Madani et al., 2017).

Collagen Adhesin-like proteins bind collagen by a “collagen hug” mechanism where the N1N2 segment “hugs” or wraps around the collagen triple helix molecule (Figure 2). A co-crystal of CNA bound in complex with the synthetic collagen peptide (GPO)_4_GPRGRT(GPO)_4_, where O is hydroxyproline, provided the insights into the molecular basis of this model. The collagen hug binding mechanism is initiated when the collagen triple helix interacts with the shallow groove on the CNA N2 domain. This interaction is low affinity, and involves polar and hydrophobic residues (Zong et al., 2005). The initial interaction leads to structural rearrangements within the N1 domain that repositions N1 closer to the N2 domain creating a “tunnel-like” structure. Finally, the C-terminal extension of the N2 domain undergoes structural changes, and inserts into the N1 domain by β-strand complementation thus forming a “latch” (Figure 2). The N1 domain of CNA interacts with the middle chain while the N2 domain interacts with the leading and trailing chains of the synthetic collagen peptide. The N1N2 linker region covers the collagen peptide and holds it in place (Zong et al., 2005; Liu et al., 2007).

The two-step binding mechanism of CNA to collagen was confirmed by atomic force microscopy studies where a moderate force (∼250 pN) was observed for the initial hydrophobic interaction between collagen and the N2 domain of CNA (Herman-Bausier et al., 2016). After binding collagen, a strong force of ∼1.2 nN was observed for the full interaction. Although B-repeats of CNA do not bind collagen directly, they act as a spring and help withstand the high mechanical stress encountered *in vivo* (Herman-Bausier et al., 2016).

Although all members of the CNA-like MSCRAMM family appear to bind collagen by a collagen hug mechanism, the proteins show differences in affinity (Table 2) and mechanistic details because of structural variations. For example, CNA has a higher affinity for the collagen triple helix than Ace (Ross et al., 2012). In contrast to the two-step mechanism used by CNA, Ace binds collagen with a rapid association and dissociation rate in a one-step binding mechanism (Rich et al., 1999; Ross et al., 2012).

#### Virulence

Most proteins in the CNA-like MSCRAMM sub-family have been shown to act as virulence factors in experimental bacterial infections. CNA-like proteins target collagen to enhance adhesion of the bacteria to host tissues in early and later stages of infection. For example, CNA is a critical virulence factor of *S. aureus* in experimental septic arthritis and osteomyelitis models and this role depends on its ability to bind collagen (Patti et al., 1994b; Elasri et al., 2002; Xu et al., 2004b). Although CNA is not required in the initial targeting of joints, it is critical for hematogenous spread of *S. aureus* leading to bone infections (Elasri et al., 2002). Additionally, more bacteria were isolated from joints of mice infected with collagen binding *cna*^+^ bacterial strains than those infected with non-collagen binding strains. Most CNA-like proteins also bind to collagen present in vegetations observed in non-bacterial thrombotic endocarditis, thus leading to infective endocarditis (Hienz et al., 1996; Nallapareddy et al., 2008; Singh et al., 2010). Ace and Acm, the enterococcal CNA-like proteins, are important virulence factors in infective endocarditis (Nallapareddy et al., 2008; Singh et al., 2010). The *ace* deletion mutant of *E. faecalis* OG1RF strain showed decreased colonization of heart valves in a mixed-infection rat endocarditis model compared to the wild type strain. Higher bacterial colony forming unit (CFU) counts were recovered from aortic valve vegetations at 4 h in mono endocarditis infection of rats with *ace* expressing *E. faecalis* OG1RF compared to the *ace* deletion mutant, indicating a role in early colonization of heart valves (Singh et al., 2010). Similarly, significantly more wild type (WT) *E. faecium* TX0082 CFUs were recovered from rat vegetations after mixed endocarditis infection compared to *acm* deletion mutant *E. faecalis* TX6051. Furthermore, Acm was also shown to enhance early adherence to heart valves (Nallapareddy et al., 2008). On the other hand, CNA’s ability to bind collagen is of limited significance in early stages of attachment to traumatized aortic valves, but like Acm and Ace (Nallapareddy et al., 2008), CNA does contribute to establishment of infection at a 24 h time point in both mono and mixed endocarditis infections of rats with *S. aureus* isolates (Hienz et al., 1996).

Cbm and Cnm are homologous *S. mutans* proteins with 78% identity in their collagen binding domains. The *cnm* gene is sufficient and necessary for primary human coronary artery endothelial cell invasion by *S. mutans* isolates as shown with Δ*cnm S. mutans* clinical isolates as well as *cnm*^+^ *Lactococcus lactis* (Abranches et al., 2011; Freires et al., 2017). The *cnm* gene also permits invasion of other non-phagocytic cells like human gingival fibroblasts and human oral keratinocytes (Miller et al., 2015). In addition, *cnm*^+^ *S. mutants* OMZ175 and *cnm*^+^ *L. lactis* outcompeted Δ*cnm S. mutans* OMZ175 and *L. lactis*, by 10 and 100-fold, respectively, in *ex vivo* bacterial adherence to aortic valve sections. Using a rabbit model of infective endocarditis, it was shown that *cnm L. lactis* mediated attachment to injured endocardium but not to the vegetations (Freires et al., 2017). Similar to Cnm, *cbm*^+^ *S. mutans* attaches to aortic valves and leads to larger vegetations formed on the impaired heart valve tissue compared to *cbm*^–^ *S. mutans*.

In addition, collagen-binding proteins have been implicated in various infections. For example, CNA has been implicated in pathogenesis of *S. aureus* keratitis (Rhem et al., 2000) and orthopedic prosthesis infections (Montanaro et al., 1999). Similarly, Cnm has been implicated in *S. mutans* cerebral hemorrhaging (Tonomura et al., 2016) and colonization of dental pulp (Nomura et al., 2016).

### M and M-Like Proteins

M-protein, described by Rebecca Lancefield almost a century ago (Lancefield, 1928), is a major cell wall-anchored protein and virulence factor present on the surface of Group A, B, and C streptococci (GAS, GBS, and GCS) (Dinkla et al., 2007; Barroso et al., 2009; Reissmann et al., 2012). There are around ∼250 known M-protein types in GAS based on sequence variation in the first 50 amino acids of the protein. Variations in the M-protein lead to strain-specific immunity and, hence, M-proteins serve as a strain typing marker (Lancefield, 1928). M proteins have multiple functions, including inhibition of phagocytosis and binding to fibrinogen, collagen, complement, and other host proteins (Metzgar and Zampolli, 2011).

#### Structure

M proteins are multi-domain proteins that adopt an elongated α- helical structure and dimerize to form helical coiled-coil structures, a structure form also seen in mammalian proteins like tropomyosin and myosin (McNamara et al., 2008; Fischetti, 2016). M-protein fibrils are ∼500 Å long and coat the surface of Group A streptococcus (Phillips et al., 1981; Fischetti, 1989). When viewed by transmission electron microscopy, M-protein appears like “fuzz on a tennis ball” (Phillips et al., 1981). All M-proteins contain a signal peptide, a hypervariable region, a less variable central domains and a highly conserved C-terminus (Figure 1; Fischetti, 1989).

The prototypic M6 protein consists of a cleavable signal sequence, A repeats, which includes the hypervariable region (HVR), B repeats, C repeats, D-region and a LPXTG motif for sortase mediated anchoring to the cell wall (Figure 1). The HVR region is the first 50 amino acids of the mature M protein and shows variation amongst the different M-proteins. The M6 A-repeat region consists of five repeats of 14 amino acids each, where the central repeats are identical and end repeats are slightly divergent (Smeesters et al., 2010; Fischetti, 2016). The B-repeat region contains five repeats, each 25 amino acid long (Fischetti, 1989, 2016). The M6 protein contains two C-repeats where each repeat is 35 residues long (Fischetti, 1989, 2016). C-repeats show higher sequence conservation compared to A- and B-repeats. Lastly, the M6 protein contains four D-repeats, each 7 amino acid long (Fischetti, 1989, 2016). Amongst the A, B, C, and D repeat regions, D-repeats show highest sequence homology to each other for any M protein (Smeesters et al., 2010). Together, A-, B-, C-, and D-repeats form the central helical rod (Fischetti, 1989, 2016, 2019; McNamara et al., 2008).

As observed in tropomyosin and myosin, the coiled-coil nature of a protein molecule comes from heptad repeats, where the first and fourth residues in the register are generally hydrophobic (Fischetti, 1989, 2016; McNamara et al., 2008). Hydrophobic residues form the core of the coiled coil and the remaining residues in the heptad repeats are generally helix promoting (McNamara et al., 2008; Fischetti, 2016). Heptad repeats found in M-proteins are not perfect, which leads to irregularities and instabilities of the coiled-coil region (McNamara et al., 2008; Macheboeuf et al., 2011). McNamara et al. found that destabilizing residues in the coiled-coil region of M1 protein promote conformational dynamics, which is required for binding of M1 protein to fibrinogen (McNamara et al., 2008; Stewart et al., 2016). These irregularities in the heptad repeats also form the basis for sub-division of the protein into A-, B-, and C-repeats (Fischetti, 2019).

Sequence and structural variations amongst M-proteins are common. Homologous recombination in M-protein leads to differences in the frequency and length of the repeats and an overall variation in size (Fischetti, 1989). As a result, A- and B- repeats are not present in all M-proteins and when present, their sizes can vary. However, all M-proteins contain C-repeats and their total number can vary from two to four (Smeesters et al., 2010). The sequence variations between M-proteins lead to functional differences and hence not all M-proteins possess all the functional capabilities described in the literature.

#### Binding Mechanism

Amongst the >250 known types of M-proteins, about 20 have been shown to bind collagen (Table 3). M-proteins bind directly to the triple helical regions of collagen (Nitsche et al., 2006; Barroso et al., 2009; Dinkla et al., 2009; Bober et al., 2011; Reissmann et al., 2012) with the exception of the M1 protein, which also interacts with the globular domain of collagen type VI (Bober et al., 2010). Rotary shadowing electron microscopy revealed that M3 protein binds collagen type IV at two different sites: one located on cyanogen bromide fragment 3 (CB3) and the other at a site 20 nm away from the 7S domain (Eble et al., 1993). CB3 is a fragment of collagen type IV that maintains its triple helix and is generated after cleavage of collagen with cyanogen bromide (Eble et al., 1993). When expressed on the surface of a heterologous non-collagen binding host (*Streptococcus gordonii* GP1221), M-proteins from GCS and Group G streptococci (GGS) enabled GP 1221 to bind to collagen type IV at the same level as GCS and GGS (Barroso et al., 2009).

**TABLE 3:** Collagen binding M-proteins.

**M-protein**	**Species**	**Collagen**	***K*_*D*_**	**References**
stG4545.0	SDSE	IV	1.8 pM	Barroso et al., 2009
stC2sk.0	SDSE	IV	3.5 pM	Barroso et al., 2009; Reissmann et al., 2012
stC5344	SDSE	IV	920 pM	Barroso et al., 2009; Reissmann et al., 2012
stG2574.0	SDSE	IV	1.2 nM	Barroso et al., 2009; Reissmann et al., 2012
stC-NSRT2.0	SDSE	IV	830 pM	Barroso et al., 2009; Reissmann et al., 2012
stG10.0	SDSE	IV	610 pM	Barroso et al., 2009; Reissmann et al., 2012
FOG (stG11.0)	SDSE	I	80 pM	Nitsche et al., 2006
		IV	6 nM	Dinkla et al., 2007; Barroso et al., 2009
M3	*S. pyogenes*	IV	5 nM	Dinkla et al., 2009; Reissmann et al., 2012
stG97	SDSE	IV	ND	Reissmann et al., 2012
stC6746	SDSE	IV	ND	Reissmann et al., 2012
M31.5	SDSE	IV	0.6 nM	Reissmann et al., 2012
M3.22	*S. pyogenes*	IV	ND	Reissmann et al., 2012
stG211.1	SDSE	IV	ND	Reissmann et al., 2012
stG120.1	SDSE	IV	ND	Reissmann et al., 2012
stG351	SDSE	IV	ND	Reissmann et al., 2012
stCQ343	SDSE	IV	ND	Reissmann et al., 2012
stG211.0	SDSE	IV	ND	Reissmann et al., 2012
stC922	SDSE	IV	ND	Reissmann et al., 2012
M55	*S. pyogenes*	IV	5 nM	Reissmann et al., 2012
M1	*S. pyogenes*	I	54 nM	Bober et al., 2011
		VI	ND	Bober et al., 2010

*SDSE stands for S. dysgalactiae* subsp. *equisimilis; ND stands for not determined.*

Peptide associated with rheumatic fever (PARF) is an eight-residue motif present in the hypervariable A region of some M- and M-like proteins (Dinkla et al., 2007; Barroso et al., 2009; Reissmann et al., 2012). Based on careful examination of multiple M-proteins from 69 isolates, a consensus sequence of the PARF motif was determined to be (A/T/E)XYLXX(L/F)N where charged amino acids are preferred at positions 2, 5, and 6, with at least one of the charged amino acids containing a basic side chain (Barroso et al., 2009; Reissmann et al., 2012). A PARF motif is required for binding of these M-proteins to collagen (Dinkla et al., 2007; Reissmann et al., 2012), as one or two substitutions of the conserved residues in the PARF motif abolishes binding to collagen type IV (Reissmann et al., 2012). However, additional data suggests that the binding of M-proteins to collagen can be more complicated and extends beyond the PARF motif. First, a series of recombinant truncated PARF-containing versions of an M-protein bind collagen with significantly different affinities (Dinkla et al., 2007). A full-length recombinant M-protein of GGS called “fibrinogen-binding protein of G streptococci” (FOG) binds to collagen type IV with a *K*_*D*_ of 6 nM, whereas a truncated FOG protein containing A- and B-repeats binds collagen type IV with 24 times higher *K*_*D*_ and a FOG protein containing the A-region only binds collagen type IV with a 200 fold higher *K*_*D*_ compared to the full length FOG protein (Dinkla et al., 2007). Similarly, a truncated recombinant FOG protein binds collagen type I with a 20 fold higher *K*_*D*_ than the full length recombinant FOG protein (Nitsche et al., 2006). Furthermore, Reissmann et al. (2012) identified M-proteins with PARF motifs that did not bind collagen type IV. Interestingly, M-proteins stG120.1, stG120.0, and stGM220 all contain the same PARF motif but only stG120.1 binds collagen type IV, while all three proteins bind fibrinogen. Moreover, the M1-protein lacks a PARF motif (Reissmann et al., 2012) but still binds to the triple helix of collagen types I and IV (Bober et al., 2011) and globular domains of collagen type VI (Bober et al., 2010).

M-proteins binding to different types of collagens can have different consequences. Binding of M-proteins to collagen type IV leads to aggregation of collagen on the surface of the bacteria (Dinkla et al., 2003b, 2007; Barroso et al., 2009), which is not observed with the interaction of collagen type I to M-protein (Barroso et al., 2009). Expression of M-protein on the surface of a heterologous host leads to collagen type IV aggregation, demonstrating that the M-protein alone is sufficient for collagen aggregation.

#### Virulence

M or M-like proteins are major virulence factors of Streptococci and their role in streptococcus pathogenesis have been reported on extensively (Oehmcke et al., 2010; Smeesters et al., 2010; Frost et al., 2017; Fischetti, 2019). In this review article, we will focus on the contribution of the M-protein:collagen interaction to the pathogenesis of streptococci. Binding of M-proteins to collagen can have two consequences: (1) mediating bacterial adhesion to connective tissues and (2) inducing collagen auto-immunity.

M-protein binding to collagen is important in the colonization of human skin by streptococci (Nitsche et al., 2006). When incubated with human dermis *ex vivo*, higher CFU counts were recovered from a GGS strain expressing FOG protein compared to a FOG-deficient strain. Incubation of the bacteria with collagen type I decreased adherence of the FOG expressing strain to human dermis, thereby also suggesting that the interaction of FOG with collagen type I enables adhesion.

Acute rheumatic fever (ARF) and rheumatic heart disease are antibody-mediated autoimmune sequelae that can develop after a streptococcal infection (Tandon et al., 2013; Carapetis et al., 2016). Binding of M-protein to collagen has been shown to be a relevant factor in developing ARF (Dinkla et al., 2003b, 2007; Barroso et al., 2009). Binding of M or M-like protein to collagen type IV can lead to production of antibodies binding the collagen molecule (Dinkla et al., 2003b, 2007; Barroso et al., 2009). Analysis of mouse sera obtained from immunization with recombinant M or M-like protein led to identification of two distinct antibody populations: anti-collagen type IV antibodies and anti-M protein antibodies. These distinct antibodies did not cross-react with each other (Dinkla et al., 2007), indicating that collagen type IV autoimmunity was not generated through molecular mimicry. In addition, sera of ARF patients contain antibodies that specifically recognize the CB3 region of collagen type IV and the collagen-binding region of the M3 protein (Dinkla et al., 2007, 2009). The N-terminal half of the protein containing the PARF motif is required for generating auto-immunity (Dinkla et al., 2007). Immunization of mice with full-length FOG led to a significantly higher titer of anti-collagen type IV antibodies compared to mice immunized with FOGB2-C2, a region of FOG that does not bind collagen (Dinkla et al., 2007). Similar results have been obtained with other M-proteins (Dinkla et al., 2007; Barroso et al., 2009). While auto-antibodies to collagen type I have not been demonstrated, given the structural similarities between the collagens, anti-collagen type IV antibodies potentially could also react with other collagen types.

## Emerging Families of Collagen-Binding Proteins

Numerous collagen-binding proteins of Gram-positive pathogens have been reported in the literature but their mechanisms of collagen binding are unclear. We have identified three emerging families of collagen-binding proteins where, although one or more than one family member binds to collagen, a clear picture of how these proteins bind to collagen is not yet available.

### von Willebrand Factor A- Domain Containing Proteins

von Willebrand factor (vWF) is a host glycoprotein found in blood, blood vessel ECM, and platelet α-granules (Manon-Jensen et al., 2016). vWF is a large modular protein that contains two binding sites for collagen located in the A1 and A3 domains. The A3 domain of vWF binds collagen types I and III whereas the A1 domain binds collagen types IV and VI (Manon-Jensen et al., 2016). Crystal structures of both A1 and A3 domains show a central β-sheet composed of six β-strands and flanked on both sides by α-helices (Huizinga et al., 1997; Emsley et al., 1998). These domains are structurally similar to the I-domain of some integrin α-chains, including the collagen-binding α1-, 2-, 10-, and 11- chains. The collagen-binding α-chain integrins also contain a metal ion-dependent adhesion site (MIDAS) important for ligand binding (Lee et al., 1995; Huizinga et al., 1997; Emsley et al., 1998).

Structural homologs of vWF A-domains, called vWA domains, have been found in minor pilus proteins that bind to ECM proteins and host cells. These pilus proteins include RrgA from *Streptococcus pneumoniae* (Izore et al., 2010), GBS104 from *Streptococcus agalactiae* (Krishnan et al., 2013), PilA from *S. agalactiae* (Konto-Ghiorghi et al., 2009; Banerjee et al., 2011), SpaC from *Corynebacterium diphtheriae* (Mandlik et al., 2007), and EbpA from *E. faecalis* (Nielsen et al., 2012). Most structural information about bacterial vWA domains comes from crystal structures of the RrgA and the GBS104 proteins (Izore et al., 2010; Krishnan et al., 2013). RrgA and GBS104 are homologs that share 51% sequence identity with each other and have a similar domain organization (Krishnan et al., 2013). Both proteins contain an N-terminal signal sequence, four D domains named D1, D2, D3, and D4, and a C-terminal sorting signal (Figure 1). The primary sequence of both D1 and D2 domains is non-contiguous, and is divided into two regions, one present in the N-terminal half and other present in the C-terminal half of the protein (Figure 1). The two regions fold back on each other to form the tertiary structure of the D1 and D2 domains. The D3 domain is inserted in between the two regions encoding the D1 and D2 domains and the D4 region is located distal to the C-terminal half of the D1-D2 domain (Figure 1). It is worth noting that while RrgA and GBS104 are structural homologs, other pilus proteins containing vWA domains like PilA have a different overall domain organization (Mandlik et al., 2007; Konto-Ghiorghi et al., 2009; Izore et al., 2010; Banerjee et al., 2011; Nielsen et al., 2012; Krishnan et al., 2013).

**FIGURE 3:**
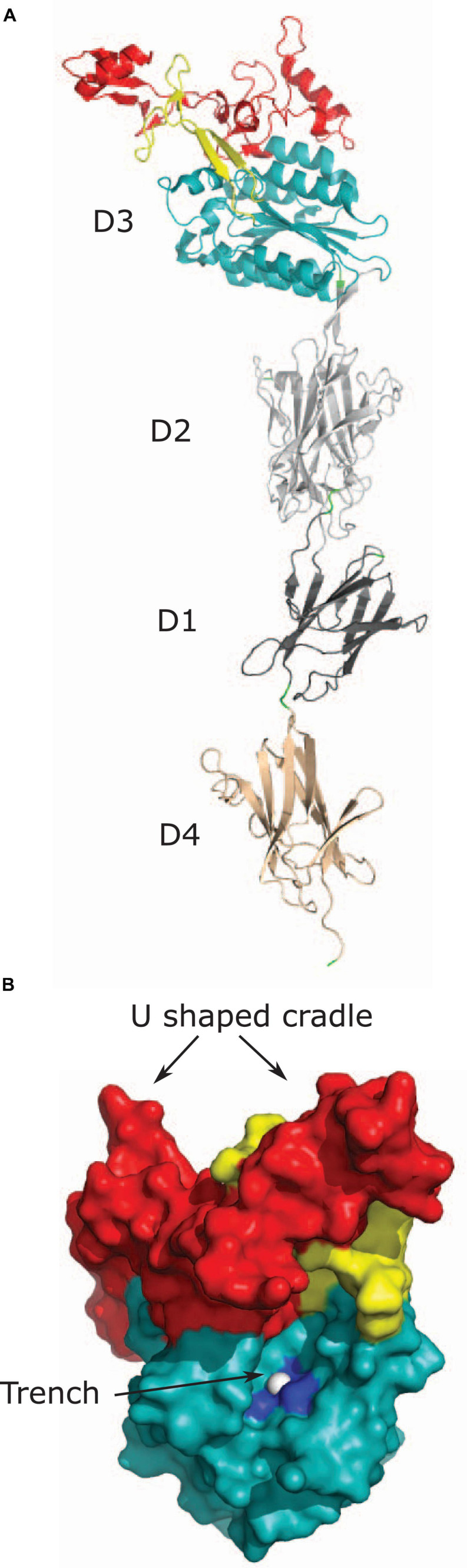
Tip pilus protein RrgA. **(A)** Tip pilus protein RrgA of *S. pneumoniae* [PDB code: 2WW8 (Izore et al., 2010)] contains a D3 domain with vWA fold (shown in teal), D1 (shown in dark gray), and D4 domains (shown in light brown) with an IgG-rev fold, and a D2 domain (shown in light gray) with a Dev-IgG fold. The collagen-binding D3 domain is positioned at the tip and contains two inserted arms in the D3 domain. First inserted arm is shown in yellow and second inserted arm is shown in red. **(B)** Figure shows two predicted collagen binding sites on the surface of D3 domain of RrgA. D3 domain, first inserted arm and second inserted arm are shown in light teal, yellow and red, respectively. The U-shaped cradle on the top of D3 domain is formed by two inserted arms. Magnesium ion present in MIDAS motif is shown as white sphere in the trench and residues interacting with magnesium ion (S234, S236, and D387) are shown in dark blue.

The D3 domains of both RrgA and GBS104 adopt a structure similar to the vWF A-domain. These D3 domains of both RrgA and GBS104 consist of a central β-sheet flanked by α-helices on both sides as seen in the vWF A-domain and the integrin I-domain (Figure 3A; Huizinga et al., 1997; Emsley et al., 1998; Izore et al., 2010; Krishnan et al., 2013). In addition, both RrgA and GBS104 have two arms inserted into the vWA-domain that are absent in the A-domains of vWF and the I-domain of integrins (Lee et al., 1995; Huizinga et al., 1997; Emsley et al., 1998; Izore et al., 2010; Krishnan et al., 2013). The first arm of RrgA contains two β-hairpins folded together to form an elongated arm (Figure 3A). The second arm of RrgA consists mostly of loops along with one short hairpin, two α-helices and loops (Figure 3A; Izore et al., 2010; Krishnan et al., 2013). The two inserted arms extend away from the core of the domain and extend the length of the protein (Figure 3A; Izore et al., 2010). The D3 domain of the two bacterial proteins also contains a MIDAS motif (Figure 3B) present in the I-domain of integrins but absent in the vWF A-domains.

Amongst the pilus proteins with a vWA-domain, RrgA, PilA, and GBS104 have been reported to bind collagen. RrgA binds collagen type I, fibronectin, and laminin (Hilleringmann et al., 2008; Moschioni et al., 2010). However, the D3 vWA domain alone was not able to bind ECM proteins (Moschioni et al., 2010). Full-length RrgA protein is required for binding (Moschioni et al., 2010). RrgA binds to collagen type I with weaker force than expected for a ligand:receptor interaction. It has been suggested that the low binding force might help the pilus adhere and detach under physiological flow conditions. However, kinetic data for RrgA and collagen type I is lacking and the suggested consequences of low binding force awaits elucidation (Becke, 2019). Although recombinant PilA has been reported to bind collagen, its role in *S. agalactiae* collagen binding is not clear (Banerjee et al., 2011; Dramsi et al., 2012). Similarly, GBS104 has been reported to bind collagen but the interaction in a solid-phase binding assay is weak and does not reach saturation indicating that the interaction of GBS104 and collagen type I may not be specific or have functional relevance (Krishnan et al., 2013).

Two different binding regions in the vWA domain-containing pilus proteins have been proposed: the vWA-domain with the MIDAS motif and the U-shaped cradle formed by the inserted arms (Figure 3B; Izore et al., 2010). Apo-crystal structure of the vWA-domain with the MIDAS motif revealed a trench-like region formed by the two inserted arms and the MIDAS motif present on the central β-sheet (Izore et al., 2010; Krishnan et al., 2013). Based on structural comparison with co-crystals of integrin α2β1 and a synthetic triple helix peptide, the trench-like region has been proposed to be the collagen binding site (Figure 3B; Emsley et al., 2000; Izore et al., 2010). The vWA-domain and the integrin I-domain undergoes conformational change during binding events and transition from a closed form to an open form. Participation of the trench-like region and a change in confirmation upon ECM binding was confirmed using an open form of the GBS104-D3 domain stabilized by a disulfide bridge. The open form of the GBS104-D3 domain alone was sufficient for binding to fibronectin, whereas the closed form of the D3 domain showed no binding (Krishnan et al., 2013). The vWA-domains of pilus proteins have considerable variation in their primary sequence, with the most variations in the inserted arms (Konto-Ghiorghi et al., 2009; Izore et al., 2010; Krishnan et al., 2013). Therefore, despite structural similarities, these pilus proteins have been suggested to bind different ligands with different affinities (Izore et al., 2010; Krishnan et al., 2013). A second binding site is the U-shape cradle formed by the two inserted arms joining together at the tip of the protein (Figure 3B). This cradle contains basic residues and has been proposed to bind negatively charged molecules like glycosaminoglycans attached to ECM proteins (Izore et al., 2010). While the vWA domain is critical for virulence (Konto-Ghiorghi et al., 2009; Nielsen et al., 2012), evidence that the vWA domain of RrgA is responsible for binding to collagen is lacking.

### Leucine Rich Repeat Containing Proteins

Leucine rich repeats (LRRs) are protein recognition motifs present in eukaryotic proteins with diverse functions (Kobe and Kajava, 2001). Small leucine rich proteoglycans (SLRPs) in mammals are an example of LRR proteins and play important roles in collagen fibrillogenesis (Kalamajski and Oldberg, 2010). LRR containing proteins have been found in some pathogenic bacteria, e.g., *Yersinia pestis*, *Listeria monocytogenes*, plants, animals, and fungi (Kobe and Kajava, 2001). Each repeat is 20–29 aa long and are often present in tandem with multiple LRRs to form an overall curved shape where β-sheets are present on the concave side and α-helices are often on the convex side (Kobe and Kajava, 2001).

Streptococcal leucine rich (Slr) protein is an LRR-containing lipoprotein present on the surface of *S. pyogenes* (Bober et al., 2011). The N-terminal half of the protein contains a 21 aa long signal sequence and 4 histidine triad motifs (Figure 1; Bober et al., 2011). The C-terminal half of the protein contains 13 leucine rich regions that form β-sheets, followed by histidine rich repeat sequences (Figure 1; Bober et al., 2011). The horseshoe shape of Slr was visible with electron microscopy (Bober et al., 2011). Orthologs of Slr have been identified in *Streptococcus suis* 05ZYH33 (1577), *Streptococcus equi* subsp. *zooepidemicus* H70 (13200), *Streptococcus dysgalactiae* subsp. *equisimilis* GGS_124 (1372), *Streptococcus agalactiae* (Blr), *Streptococcus uberis* 0140J (1212), and *Streptococcus suis* 2 (HtpsC) (Waldemarsson et al., 2006; Plumptre et al., 2012; Li et al., 2015). Slr has been demonstrated to bind collagen directly with a *K*_*D*_ of 12 nM (Waldemarsson et al., 2006). Interestingly, HtpsC has been reported to bind laminin and fibronectin, but HtpsC did not bind collagen type I (Li et al., 2015).

The extracellular matrix protein (Emp) of *S. aureus* is 340 aa long secreted protein with a 26 aa long signal peptide at the N-terminus. Emp binds collagen type I with a *K*_*D*_ of 27 nM. Emp is structurally intriguing, as Emp is not predicted to be multi-domained. When viewed through a transmission electron microscope, the Emp monomer was revealed to form a horseshoe-type structure with an 8 nm diameter. Interestingly, even though it lacks leucine repeats, structure prediction through I-TASSER identified leucine rich repeat proteins as the top ten structural analogs (Geraci et al., 2017).

While molecular details of Slr and Emp binding to collagen have not been studied beyond the confirmation of their interaction, their intriguing overall structural similarities leads the way for postulating an emerging LRR-containing or LRR like protein family that binds collagen. Given that several human LRR proteins [e.g., decorin (Schönherr et al., 1995), fibromodulin (Font et al., 1998)] interact with collagen, it is not surprising that bacterial LRR proteins bind collagen. Additional studies are needed to determine the residues that mediate the interaction and determine similarities of those interactions with host LRR proteins and collagen.

### Sgo0707 N1-Domain Containing Proteins

**FIGURE 4:**
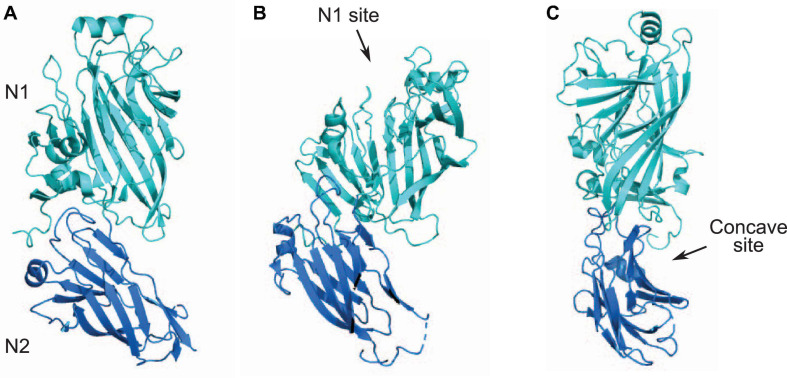
N1N2 domains of Sgo0707. **(A)** N1 and N2 domains of Sgo0707 from *S. gordonii* [PDB code: 4IGB (Nylander et al., 2013)] are shown in light teal and dark blue, respectively. **(B)** Figure shows predicted collagen binding site present in the N1 domain. **(C)** Figure shows second predicted collagen binding site formed the loops of the N1 domain and a β-sheet of the N2 domain. This site forms a concave surface where collagen can dock.

Streptococci express multiple surface proteins that have been reported to bind collagen (Avilés-Reyes et al., 2017). One emerging family of collagen-binding proteins in Streptococci is related to the N1-domain of Sgo0707 protein from *S. gordonii*. The Sgo0707 protein, which has been shown to bind collagen, contains a N-terminal signal sequence, a 419 aa long N-terminal region, eight repeats of 84 aa, five repeats of 88 aa, a unique domain, an LPXTG cell wall sorting signal and a transmembrane helix (Figure 1; Nylander et al., 2013). The 419 aa long N-terminal region is divided into two domains: N1 and N2 (Figure 1). Both the N1 and N2 domains adopt a β-sandwich with anti-parallel β-sheets (Nylander et al., 2013), where β-sheet 1 contains nine β-strands and β-sheet 2 contains eight strands (Figure 4A). The N1 domain also contains two small sub-domains A and B. The N2 domain consists of two β-sheets of five β-strands and a third small sheet of three strands and adopts a DeV-IgG fold also observed in the N1N2 domains of CNA (Nylander et al., 2013).

A search of proteins with similar N1 domains identified the variable domains in two Ag I/II family proteins; SpaP from *S. mutans* and SspB from *S. gordonii*. Both these domains are predicted to adopt a similar structure despite having only 10% sequence identity to N1 of Sgo0707 (Forsgren et al., 2009; Larson et al., 2010; Nylander et al., 2013). These proteins have a different domain organization than Sgo0707, with an N-terminal signal sequence, alanine rich repeats, a variable domain, proline rich repeats, a C-terminal domain, and an LPXTG cell wall sorting signal (Forsgren et al., 2009; Larson et al., 2010). All three proteins form an extended confirmation with the putative collagen binding domain (N-region of Sgo0707 and variable domain of SpaP and SspB) predicted to be located at the tip of the protein (Forsgren et al., 2009; Larson et al., 2010; Nylander et al., 2013).

Docking of the collagen triple helix to the Sgo0707 N1N2 domain identified two different potential binding sites (Figures 4B,C). The first binding site is on top of the N1 domain in the open cleft formed by the two subdomains in the N1 domain (Figure 4B). This site has a higher negative surface potential compared to the SspB and SpaP proteins, and lacks the metal ion located in the cleft found in both of the Ag I/II proteins (Forsgren et al., 2009; Larson et al., 2010; Nylander et al., 2013). A second putative collagen-binding site is formed by the loops of the N1 domain and a β-sheet of the N2 domain, which together form a concave surface where collagen can dock (Figure 4C; Nylander et al., 2013). The concave site consists of mostly non-polar residues (Nylander et al., 2013).

All three proteins (Sgo0707, SspB, and SpaP) have been implicated in collagen binding. Binding of the three proteins to collagen type I was shown in a bacterial adhesion assay using deletion mutants (Love et al., 1997, 2000; Nylander et al., 2013). Deletion of the *sgo0707* gene in *S. gordonii* DL1 decreased collagen type I binding by 40% compared to the WT strain (Nylander et al., 2013). Similarly, an isogenic deletion mutant of the *sspB* gene in *S. gordonii* and of the *spaP* gene in *S. mutans* showed decreased binding to collagen type I compared to WT strains (Love et al., 1997, 2000). Additionally, binding of *S. gordonii* DL1 to collagen type I in a bacterial adhesion assay was inhibited by recombinant N-region of Sgo0707, thus narrowing down the N-region as the collagen binding partner (Nylander et al., 2013). While the three proteins have been implicated in collagen binding, their direct binding to collagen has not been demonstrated. The three proteins only share structurally similar N1-domains as both SspB and SpaP lack the N2 domain found in Sgo0707 (Love et al., 1997, 2000; Nylander et al., 2013). Do the three proteins bind collagen at the cleft on top of the N1-domain? Further studies are required to narrow down the collagen-binding site in these proteins and to determine if they form a structural family of proteins that bind collagen.

## Concluding Remarks

Gram-positive pathogens utilize their interactions with the ECM for tissue colonization and to establish infections in the host. Molecular insight into these interactions can pave the way for the design of novel anti-infectives. However, studies of collagen-binding proteins in Gram-positive pathogens are in their infancy and do not provide a complete picture of the different binding mechanisms involved. Further structural studies are required to fully understand the molecular basis for the interaction between bacterial collagen-binding proteins and the triple helix of collagen. In particular, the interaction of emerging collagen-binding protein families with collagen needs to be further characterized using biochemical and microbiological techniques to determine which family members bind collagen.

Mammalian proteins containing a collagen-like region play a role in host defense. Collagen-binding host proteins, e.g., the LRR proteoglycan decorin, bind soluble host defense collagens (Krumdieck et al., 1992). Interaction of bacterial collagen-binding proteins with soluble defense collagens can provide an opportunity for pathogens to evade the host immune response. CNA binds C1q, a complement protein and collagen (Kang et al., 2013). The classical complement pathway is initiated upon recognition of pathogen-bound antibodies by the C1 complex, which consists of C1q, C1r, and C1s. C1q protein contains the globular recognition domain and binds pathogen-bound antibodies. C1r and C1s are proteases that are required for the complement cascade. C1r and C1s bind the collagen-like stalk of C1q (Mortensen et al., 2017). CNA uses its interaction with C1q for immune evasion by interfering with the interaction between C1r and C1q and thus deactivating the C1 complex (Kang et al., 2013).

Interaction of collagen-binding bacterial proteins with other host proteins containing collagen like-regions, especially soluble human defense collagens, is an understudied area. While acknowledging that not all collagen-binding proteins will bind soluble defense collagens and vice versa, future studies focusing on the interaction between bacterial collagen binding proteins and host defense collagens will lead to a better understanding of the pathogenic mechanisms utilized by Gram-positive bacteria.

## Author Contributions

SA and JG wrote the manuscript. SA and MH edited the manuscript. All authors contributed to the article and approved the submitted version.

## Conflict of Interest

The authors declare that the research was conducted in the absence of any commercial or financial relationships that could be construed as a potential conflict of interest.

## References

[B1] Abranches J. Miller J. H. Martinez A. R. Simpson-Haidaris P. J. Burne R. A. Lemos J. A. (2011). The collagen-binding protein Cnm is required for Streptococcus mutans adherence to and intracellular invasion of human coronary artery endothelial cells. *Infect. Immun.* 79 2277–2284. 10.1128/iai.00767-10 21422186 PMC3125845

[B2] Arvanitidis A. Karsdal M. A. (2016). “Type XV Collagen,” in *Biochemistry of Collagens, Laminins and Elastin*, Chap. 15, ed. Karsdal M. A. (Cambridge, MA: Academic Press), 97–99.

[B3] Avilés-Reyes A. Miller J. H. Lemos J. A. Abranches J. (2017). Collagen-binding proteins of Streptococcus mutans and related streptococci. *Mol. Oral. Microbiol.* 32 89–106. 10.1111/omi.12158 26991416 PMC5025393

[B4] Bager C. L. Karsdal M. A. (2016). “Type XVIII Collagen,” in *Biochemistry of Collagens, Laminins and Elastin*, Chap. 18, ed. Karsdal M. A. (Cambridge, MA: Academic Press), 113–121.

[B5] Banerjee A. Kim B. J. Carmona E. M. Cutting A. S. Gurney M. A. Carlos C. (2011). Bacterial Pili exploit integrin machinery to promote immune activation and efficient blood-brain barrier penetration. *Nat. Commun.* 2:462. 10.1038/ncomms1474 21897373 PMC3195231

[B6] Barroso V. Rohde M. Davies M. R. Gillen C. M. Nitsche-Schmitz D. P. Dinkla K. (2009). Identification of active variants of PARF in human pathogenic group C and group G streptococci leads to an amended description of its consensus motif. *Int. J. Med. Microbiol.* 299 547–553. 10.1016/j.ijmm.2009.04.004 19520603

[B7] Becke T. (2019). *Streptococcus Pneumoniae TIGR4 Pilus-1 Biomechanical Aspects of Adhesion During Interaction With Host Extracellular Matrix Proteins Fibronectin and Collagen I.* München: Technische Universität München.

[B8] Bober M. Enochsson C. Collin M. Mörgelin M. (2010). Collagen VI is a subepithelial adhesive target for human respiratory tract pathogens. *J. Innate Immun.* 2 160–166. 10.1159/000232587 20375633

[B9] Bober M. Mörgelin M. Olin A. I. von Pawel-Rammingen U. Collin M. (2011). The membrane bound LRR lipoprotein Slr, and the cell wall-anchored M1 protein from *Streptococcus pyogenes* both interact with type I collagen. *PLoS One* 6:e20345. 10.1371/journal.pone.0020345 21655249 PMC3105044

[B10] Boudko S. P. Bächinger H. P. (2016). Structural insight for chain selection and stagger control in collagen. *Sci. Rep.* 6:37831. 10.1038/srep37831 27897211 PMC5126661

[B11] Burgeson R. E. Nimni M. E. (1992). Collagen types. molecular structure and tissue distribution. *Clin. Orthop. Relat. Res.* 282 250–272. 1516320

[B12] Carapetis J. R. Beaton A. Cunningham M. W. Guilherme L. Karthikeyan G. Mayosi B. M. (2016). Acute rheumatic fever and rheumatic heart disease. *Nat. Rev. Dis. Primers* 2:15084. 10.1038/nrdp.2015.84 27188830 PMC5810582

[B13] Carret G. Emonard H. Fardel G. Druguet M. Herbage D. Flandrois J. P. (1985). Gelatin and collagen binding to *Staphylococcus aureus* strains. *Ann. Inst. Pasteur. Microbiol. (1985)* 136a 241–245. 10.1016/s0769-2609(85)80063-6 4004151

[B14] Casals C. Campanero-Rhodes M. A. García-Fojeda B. Solís D. (2018). The role of collectins and galectins in lung innate immune defense. *Front. Immunol.* 9:1998. 10.3389/fimmu.2018.01998 30233589 PMC6131309

[B15] Casals C. García-Fojeda B. Minutti C. M. (2019). Soluble defense collagens: sweeping up immune threats. *Mol. Immunol.* 112 291–304. 10.1016/j.molimm.2019.06.007 31228661

[B16] Deivanayagam C. C. Rich R. L. Carson M. Owens R. T. Danthuluri S. Bice T. (2000). Novel fold and assembly of the repetitive B region of the *Staphylococcus aureus* collagen-binding surface protein. *Structure* 8 67–78. 10673425 10.1016/s0969-2126(00)00081-2

[B17] Desvaux M. Dumas E. Chafsey I. Hébraud M. (2006). Protein cell surface display in Gram-positive bacteria: from single protein to macromolecular protein structure. *FEMS Microbiol. Lett.* 256 1–15. 10.1111/j.1574-6968.2006.00122.x 16487313

[B18] Dinkla K. Nitsche-Schmitz D. P. Barroso V. Reissmann S. Johansson H. M. Frick I. M. (2007). Identification of a streptococcal octapeptide motif involved in acute rheumatic fever. *J. Biol. Chem.* 282 18686–18693. 10.1074/jbc.M701047200 17452321

[B19] Dinkla K. Rohde M. Jansen W. T. Carapetis J. R. Chhatwal G. S. Talay S. R. (2003a). *Streptococcus pyogenes* recruits collagen via surface-bound fibronectin: a novel colonization and immune evasion mechanism. *Mol. Microbiol.* 47 861–869. 10.1046/j.1365-2958.2003.03352.x 12535082

[B20] Dinkla K. Rohde M. Jansen W. T. M. Kaplan E. L. Chhatwal G. S. Talay S. R. (2003b). Rheumatic fever-associated *Streptococcus pyogenes* isolates aggregate collagen. *J. Clin. Invest.* 111 1905–1912. 10.1172/JCI17247 12813026 PMC161421

[B21] Dinkla K. Talay S. R. Mörgelin M. Graham R. M. A. Rohde M. Nitsche-Schmitz D. P. (2009). Crucial role of the CB3-region of collagen IV in PARF-induced acute rheumatic fever. *PLoS One* 4:e4666. 10.1371/journal.pone.0004666 19252743 PMC2646144

[B22] Dramsi S. Morello E. Poyart C. Trieu-Cuot P. (2012). Epidemiologically and clinically relevant Group B Streptococcus isolates do not bind collagen but display enhanced binding to human fibrinogen. *Microbes Infect.* 14 1044–1048. 10.1016/j.micinf.2012.07.004 22841805

[B23] Duarte A. S. Correia A. Esteves A. C. (2016). Bacterial collagenases – A review. *Crit. Rev. Microbiol.* 42 106–126. 10.3109/1040841X.2014.904270 24754251

[B24] Eble J. A. Golbik R. Mann K. Kühn K. (1993). The alpha 1 beta 1 integrin recognition site of the basement membrane collagen molecule [alpha 1(IV)]2 alpha 2(IV). *Embo J.* 12 4795–4802. 8223488 10.1002/j.1460-2075.1993.tb06168.x PMC413926

[B25] Elasri M. O. Thomas J. R. Skinner R. A. Blevins J. S. Beenken K. E. Nelson C. L. (2002). *Staphylococcus aureus* collagen adhesin contributes to the pathogenesis of osteomyelitis. *Bone* 30 275–280. 11792597 10.1016/s8756-3282(01)00632-9

[B26] Emsley J. Cruz M. Handin R. Liddington R. (1998). Crystal structure of the von Willebrand Factor A1 domain and implications for the binding of platelet glycoprotein Ib. *J. Biol. Chem.* 273 10396–10401. 10.1074/jbc.273.17.10396 9553097

[B27] Emsley J. Knight C. G. Farndale R. W. Barnes M. J. Liddington R. C. (2000). Structural basis of collagen recognition by integrin alpha2beta1. *Cell* 101 47–56. 10.1016/s0092-8674(00)80622-4 10778855

[B28] Fischetti V. A. (1989). Streptococcal M protein: molecular design and biological behavior. *Clin. Microbiol. Rev.* 2 285–314. 10.1128/cmr.2.3.285 2670192 PMC358122

[B29] Fischetti V. A. (2016). *M Protein and Other Surface Proteins on Streptococci.* Oklahoma: University of Oklahoma Helath Sciences Center. 26866233

[B30] Fischetti V. A. (2019). Surface proteins on gram-positive bacteria. *Microbiol. Spectr.* 7 GPP3-0012-2018. 10.1128/microbiolspec.GPP3-0012-2018 31373270 PMC6684298

[B31] Fitzgerald J. Holden P. Hansen U. (2013). The expanded collagen VI family: new chains and new questions. *Connect Tissue Res.* 54 345–350. 10.3109/03008207.2013.822865 23869615 PMC5248970

[B32] Font B. Eichenberger D. Goldschmidt D. Boutillon M. M. Hulmes D. J. (1998). Structural requirements for fibromodulin binding to collagen and the control of type I collagen fibrillogenesis–critical roles for disulphide bonding and the C-terminal region. *Eur. J. Biochem.* 254 580–587. 10.1046/j.1432-1327.1998.2540580.x 9688269

[B33] Forsgren N. Lamont R. J. Persson K. (2009). Crystal structure of the variable domain of the *Streptococcus gordonii* surface protein SspB. *Protein Sci.* 18 1896–1905. 10.1002/pro.200 19609934 PMC2777364

[B34] Foster T. J. (2019). The MSCRAMM family of cell-wall-anchored surface proteins of gram-positive cocci. *Trends Microbiol.* 27 927–941. 10.1016/j.tim.2019.06.007 31375310

[B35] Foster T. J. Geoghegan J. A. Ganesh V. K. Höök M. (2014). Adhesion, invasion and evasion: the many functions of the surface proteins of *Staphylococcus aureus*. *Nat. Rev. Microbiol.* 12 49–62. 10.1038/nrmicro3161 24336184 PMC5708296

[B36] Frantz C. Stewart K. M. Weaver V. M. (2010). The extracellular matrix at a glance. *J. Cell Sci.* 123(Pt 24), 4195–4200. 10.1242/jcs.023820 21123617 PMC2995612

[B37] Fraser D. A. Tenner A. J. (2008). Directing an appropriate immune response: the role of defense collagens and other soluble pattern recognition molecules. *Curr. Drug Targets* 9 113–122. 10.2174/138945008783502476 18288962

[B38] Freires I. A. Avilés-Reyes A. Kitten T. Simpson-Haidaris P. J. Swartz M. Knight P. A. (2017). Heterologous expression of *Streptococcus mutans* Cnm in *Lactococcus lactis* promotes intracellular invasion, adhesion to human cardiac tissues and virulence. *Virulence* 8 18–29. 10.1080/21505594.2016.1195538 27260618 PMC5963194

[B39] Frost H. R. Sanderson-Smith M. Walker M. Botteaux A. Smeesters P. R. (2017). Group A streptococcal M-like proteins: from pathogenesis to vaccine potential. *FEMS Microbiol. Rev.* 42 193–204. 10.1093/femsre/fux057 29228173

[B40] Gebauer J. M. Kobbe B. Paulsson M. Wagener R. (2016). Structure, evolution and expression of collagen XXVIII: lessons from the zebrafish. *Matrix Biol.* 49 106–119. 10.1016/j.matbio.2015.07.001 26235539

[B41] Geraci J. Neubauer S. Pöllath C. Hansen U. Rizzo F. Krafft C. (2017). The *Staphylococcus aureus* extracellular matrix protein (Emp) has a fibrous structure and binds to different extracellular matrices. *Sci. Rep.* 7:13665. 10.1038/s41598-017-14168-4 29057978 PMC5651841

[B42] Gonçalves T. J. M. Boutillon F. Lefebvre S. Goffin V. Iwatsubo T. Wakabayashi T. (2019). Collagen XXV promotes myoblast fusion during myogenic differentiation and muscle formation. *Sci. Rep.* 9:5878. 10.1038/s41598-019-42296-6 30971718 PMC6458142

[B43] Gudmann N. S. Karsdal M. A. (2016). “Type II collagen,” in *Biochemistry of Collagens, Laminins and Elastin*, Chap. 2, ed. Karsdal M. A. (Cambridge, MA: Academic Press), 13–20.

[B44] Hansen N. U. B. Karsdal M. A. (2016). “Type VIII collagen,” in *Biochemistry of Collagens, Laminins and Elastin*, Chap. 8, ed. Karsdal M. A. (Cambridge, MA: Academic Press), 61–65.

[B45] Harrington D. J. (1996). Bacterial collagenases and collagen-degrading enzymes and their potential role in human disease. *Infect. Immun.* 64 1885–1891. 10.1128/iai.64.6.1885-1891.1996 8675283 PMC174012

[B46] He Y. Karsdal M. A. (2016). “Type IX collagen,” in *Biochemistry of Collagens, Laminins and Elastin*, Chap. 9, ed. Karsdal M. A. (Cambridge, MA: Academic Press), 67–71.

[B47] Henriksen K. Karsdal M. A. (2016). “Type I collagen,” in *Biochemistry of Collagens, Laminins and Elastin*, Chap. 1, ed. Karsdal M. A. (Cambridge, MA: Academic Press), 1–11.

[B48] Herman-Bausier P. Valotteau C. Pietrocola G. Rindi S. Alsteens D. Foster T. J. (2016). Mechanical strength and inhibition of the *Staphylococcus aureus* collagen-binding protein Cna. *mBio* 7:e1529–16. 10.1128/mBio.01529-16 27795393 PMC5080380

[B49] Hienz S. A. Schennings T. Heimdahl A. Flock J. I. (1996). Collagen binding of *Staphylococcus aureus* is a virulence factor in experimental endocarditis. *J. Infect. Dis.* 174 83–88. 8656018 10.1093/infdis/174.1.83

[B50] Hilleringmann M. Giusti F. Baudner B. C. Masignani V. Covacci A. Rappuoli R. (2008). Pneumococcal Pili are composed of protofilaments exposing adhesive clusters of Rrg A. *PLoS Pathog.* 4:e1000026. 10.1371/journal.ppat.1000026 18369475 PMC2265430

[B51] Hohenester E. Yurchenco P. D. (2013). Laminins in basement membrane assembly. *Cell Adh. Migr.* 7 56–63. 10.4161/cam.21831 23076216 PMC3544787

[B52] Holderbaum D. Spech R. A. Ehrhart L. A. (1985). Specific binding of collagen to *Staphylococcus aureus*. *Coll. Relat. Res.* 5 261–271. 4042603 10.1016/s0174-173x(85)80016-9

[B53] Holmes D. F. Lu Y. Starborg T. Kadler K. E. (2018). “Collagen fibril assembly and function,” in *Current Topics in Developmental Biology*, Chap. Three, eds Litscher E. S. Wassarman P. M. (Cambridge, MA: Academic Press), 107–142. 10.1016/bs.ctdb.2018.02.004 29853175

[B54] Huizinga E. G. van der Plas R. M. Kroon J. Sixma J. J. Gros P. (1997). Crystal structure of the A3 domain of human von Willebrand factor: implications for collagen binding. *Structure* 5 1147–1156. 10.1016/s0969-2126(97)00266-9 9331419

[B55] Izore T. Contreras-Martel C. El Mortaji L. Manzano C. Terrasse R. Vernet T. (2010). Structural basis of host cell recognition by the pilus adhesin from *Streptococcus pneumoniae*. *Structure* 18 106–115. 10.1016/j.str.2009.10.019 20152157

[B56] Izu Y. Adams S. M. Connizzo B. K. Beason D. P. Soslowsky L. J. Koch M. (2020). Collagen XII mediated cellular and extracellular mechanisms regulate establishment of tendon structure and function. *Matrix Biol.* 10.1016/j.matbio.2020.10.004 [Epub ahead of print]. 33096204 PMC7870578

[B57] Kadler K. E. (2017). Fell muir lecture: collagen fibril formation *in vitro* and in vivo. *Int. J. Exp. Pathol.* 98 4–16. 10.1111/iep.12224 28508516 PMC5447863

[B58] Kadler K. E. Baldock C. Bella J. Boot-Handford R. P. (2007). Collagens at a glance. *J. Cell Sci.* 120:1955. 10.1242/jcs.03453 17550969

[B59] Kalamajski S. Oldberg Å (2010). The role of small leucine-rich proteoglycans in collagen fibrillogenesis. *Matrix Biol.* 29 248–253. 10.1016/j.matbio.2010.01.001 20080181

[B60] Kang M. Ko Y. P. Liang X. Ross C. L. Liu Q. Murray B. E. (2013). Collagen-binding microbial surface components recognizing adhesive matrix molecule (MSCRAMM) of Gram-positive bacteria inhibit complement activation via the classical pathway. *J. Biol. Chem.* 288 20520–20531. 10.1074/jbc.M113.454462 23720782 PMC3711317

[B61] Kehlet S. N. Karsdal M. A. (2016). “Type XXI collagen,” in *Biochemistry of Collagens, Laminins and Elastin*, Chap. 21, ed. Karsdal M. A. (Cambridge, MA: Academic Press), 131–133.

[B62] Kobe B. Kajava A. V. (2001). The leucine-rich repeat as a protein recognition motif. *Curr. Opin. Struct. Biol.* 11 725–732. 10.1016/S0959-440X(01)00266-4 11751054

[B63] Konto-Ghiorghi Y. Mairey E. Mallet A. Duménil G. Caliot E. Trieu-Cuot P. (2009). Dual role for pilus in adherence to epithelial cells and biofilm formation in *Streptococcus agalactiae*. *PLoS Pathog.* 5:e1000422. 10.1371/journal.ppat.1000422 19424490 PMC2674936

[B64] Krishnan V. Dwivedi P. Kim B. J. Samal A. Macon K. Ma X. (2013). Structure of *Streptococcus agalactiae* tip pilin GBS104: a model for GBS pili assembly and host interactions. *Acta Crystallogr. D Biol. Crystallogr.* 69(Pt 6), 1073–1089. 10.1107/s0907444913004642 23695252 PMC3663123

[B65] Krumdieck R. Höök M. Rosenberg L. C. Volanakis J. E. (1992). The proteoglycan decorin binds C1q and inhibits the activity of the C1 complex. *J. Immunol.* 149 3695–3701. 1431141

[B66] Lancefield R. C. (1928). The antigenic complex of *Streptococcus haemolyticus* : I. demonstration of a type-specific substance in extracts of *Streptococcus haemolyticus*. *J. Exp. Med.* 47 91–103. 10.1084/jem.47.1.91 19869404 PMC2131344

[B67] Lannergard J. Frykberg L. Guss B. (2003). CNE, a collagen-binding protein of *Streptococcus equi*. *FEMS Microbiol. Lett.* 222 69–74. 12757948 10.1016/S0378-1097(03)00222-2

[B68] Larson M. R. Rajashankar K. R. Patel M. H. Robinette R. A. Crowley P. J. Michalek S. (2010). Elongated fibrillar structure of a streptococcal adhesin assembled by the high-affinity association of alpha- and PPII-helices. *Proc. Natl. Acad. Sci. U.S.A.* 107 5983–5988. 10.1073/pnas.0912293107 20231452 PMC2851892

[B69] Lee J. O. Rieu P. Arnaout M. A. Liddington R. (1995). Crystal structure of the A domain from the alpha subunit of integrin CR3 (CD11b/CD18). *Cell* 80 631–638. 10.1016/0092-8674(95)90517-0 7867070

[B70] Leeming D. J. Karsdal M. A. (2016). “Type V collagen,” in *Biochemistry of Collagens, Laminins and Elastin*, Chap. 5, ed. Karsdal M. A. (Cambridge, MA: Academic Press), 43–48.

[B71] Li M. Shao Z.-Q. Guo Y. Wang L. Hou T. Hu D. (2015). The type II histidine triad protein HtpsC is a novel adhesion with the involvement of *Streptococcus suis* virulence. *Virulence* 6 631–641. 10.1080/21505594.2015.1056971 26151575 PMC4720241

[B72] Liu Q. Ponnuraj K. Xu Y. Ganesh V. K. Sillanpaa J. Murray B. E. (2007). The *Enterococcus faecalis* MSCRAMM ACE binds its ligand by the Collagen Hug model. *J. Biol. Chem.* 282 19629–19637. 10.1074/jbc.M611137200 17392280

[B73] Lizcano A. Sanchez C. J. Orihuela C. J. (2012). A role for glycosylated serine-rich repeat proteins in Gram-positive bacterial pathogenesis. *Mol. Oral Microbiol.* 27 257–269. 10.1111/j.2041-1014.2012.00653.x 22759311 PMC3390760

[B74] Love R. M. McMillan M. D. Jenkinson H. F. (1997). Invasion of dentinal tubules by oral streptococci is associated with collagen recognition mediated by the antigen I/II family of polypeptides. *Infect. Immun.* 65 5157–5164. 10.1128/iai.65.12.5157-5164.1997 9393810 PMC175743

[B75] Love R. M. McMillan M. D. Park Y. Jenkinson H. F. (2000). Coinvasion of dentinal tubules by *Porphyromonas gingivalis* and *Streptococcus gordonii* depends upon binding specificity of streptococcal antigen I/II adhesin. *Infect. Immun.* 68 1359–1365. 10.1128/iai.68.3.1359-1365.2000 10678948 PMC97289

[B76] Luo Y. Sinkeviciute D. He Y. Karsdal M. Henrotin Y. Mobasheri A. (2017). The minor collagens in articular cartilage. *Protein Cell* 8 560–572. 10.1007/s13238-017-0377-7 28213717 PMC5546929

[B77] Luo Y. Y. Karsdal M. A. (2016). “Type XI collagen,” in *Biochemistry of Collagens, Laminins and Elastin*, Chap. 11, ed. Karsdal M. A. (Cambridge, MA: Academic Press), 77–80.

[B78] Macheboeuf P. Buffalo C. Fu C.-Y. Zinkernagel A. S. Cole J. N. Johnson J. E. (2011). Streptococcal M1 protein constructs a pathological host fibrinogen network. *Nature* 472 64–68. 10.1038/nature09967 21475196 PMC3268815

[B79] Madani A. Garakani K. Mofrad M. R. K. (2017). Molecular mechanics of *Staphylococcus aureus* adhesin, CNA, and the inhibition of bacterial adhesion by stretching collagen. *PLoS One* 12:e0179601. 10.1371/journal.pone.0179601 28665944 PMC5493303

[B80] Mandlik A. Swierczynski A. Das A. Ton-That H. (2007). *Corynebacterium diphtheriae* employs specific minor pilins to target human pharyngeal epithelial cells. *Mol. Microbiol.* 64 111–124. 10.1111/j.1365-2958.2007.05630.x 17376076 PMC2844904

[B81] Manon-Jensen T. Karsdal M. A. (2016). “Type XIV collagen,” in *Biochemistry of Collagens, Laminins and Elastin*, Chap. 14, ed. Karsdal M. A. (Cambridge, MA: Academic Press), 93–95.

[B82] Manon-Jensen T. Kjeld N. G. Karsdal M. A. (2016). Collagen-mediated hemostasis. *J. Thromb. Haemost.* 14 438–448. 10.1111/jth.13249 26749406

[B83] McNamara C. Zinkernagel A. S. Macheboeuf P. Cunningham M. W. Nizet V. Ghosh P. (2008). Coiled-coil irregularities and instabilities in group a *Streptococcus* M1 are required for virulence. *Science* 319:1405. 10.1126/science.1154470 18323455 PMC2288698

[B84] Metzgar D. Zampolli A. (2011). The M protein of group A *Streptococcus* is a key virulence factor and a clinically relevant strain identification marker. *Virulence* 2 402–412. 10.4161/viru.2.5.16342 21852752

[B85] Miller J. H. Avilés-Reyes A. Scott-Anne K. Gregoire S. Watson G. E. Sampson E. (2015). The collagen binding protein Cnm contributes to oral colonization and cariogenicity of *Streptococcus mutans* OMZ175. *Infect. Immun.* 83:2001. 10.1128/IAI.03022-14 25733523 PMC4399078

[B86] Montanaro L. Arciola C. R. Baldassarri L. Borsetti E. (1999). Presence and expression of collagen adhesin gene (cna) and slime production in *Staphylococcus aureus* strains from orthopaedic prosthesis infections. *Biomaterials* 20 1945–1949. 10.1016/s0142-9612(99)00099-x 10514072

[B87] Mortensen J. H. Karsdal M. A. (2016). “Type VII collagen,” in *Biochemistry of Collagens, Laminins and Elastin*, Chap. 7, ed. Karsdal M. A. (Cambridge, MA: Academic Press), 57–60.

[B88] Mortensen S. A. Sander B. Jensen R. K. Pedersen J. S. Golas M. M. Jensenius J. C. (2017). Structure and activation of C1, the complex initiating the classical pathway of the complement cascade. *Proc. Natl. Acad. Sci. U.S.A.* 114 986–991. 28104818 10.1073/pnas.1616998114 PMC5293073

[B89] Moschioni M. Emolo C. Biagini M. Maccari S. Pansegrau W. Donati C. (2010). The two variants of the *Streptococcus pneumoniae* pilus 1 RrgA adhesin retain the same function and elicit cross-protection in vivo. *Infect. Immun.* 78 5033–5042. 10.1128/iai.00601-10 20823200 PMC2981310

[B90] Nallapareddy S. R. Qin X. Weinstock G. M. Höök M. Murray B. E. (2000). *Enterococcus faecalis* adhesin, ace, mediates attachment to extracellular matrix proteins collagen type IV and laminin as well as collagen type I. *Infect. Immun.* 68 5218–5224. 10.1128/iai.68.9.5218-5224.2000 10948147 PMC101781

[B91] Nallapareddy S. R. Singh K. V. Murray B. E. (2008). Contribution of the collagen adhesin acm to pathogenesis of *Enterococcus faecium* in experimental endocarditis. *Infect. Immun.* 76 4120–4128. 10.1128/iai.00376-08 18591236 PMC2519397

[B92] Nallapareddy S. R. Weinstock G. M. Murray B. E. (2003). Clinical isolates of *Enterococcus faecium* exhibit strain-specific collagen binding mediated by Acm, a new member of the MSCRAMM family. *Mol. Microbiol.* 47 1733–1747. 12622825 10.1046/j.1365-2958.2003.03417.x

[B93] Nielsen H. V. Guiton P. S. Kline K. A. Port G. C. Pinkner J. S. Neiers F. (2012). The metal ion-dependent adhesion site motif of the *Enterococcus faecalis* EbpA pilin mediates pilus function in catheter-associated urinary tract infection. *mBio* 3 e177–12. 10.1128/mBio.00177-12 22829678 PMC3419518

[B94] Nielsen M. J. Karsdal M. A. (2016a). “Type III collagen,” in *Biochemistry of Collagens, Laminins and Elastin*, Chap. 3, ed. Karsdal M. A. (Cambridge, MA: Academic Press), 21–30.

[B95] Nielsen S. H. Karsdal M. A. (2016b). “Type XIX collagen,” in *Biochemistry of Collagens, Laminins and Elastin*, Chap. 19, ed. Karsdal M. A. (Cambridge, MA: Academic Press), 123–125.

[B96] Nitsche D. P. Johansson H. M. Frick I. M. Mörgelin M. (2006). Streptococcal protein FOG, a novel matrix adhesin interacting with collagen I in vivo. *J. Biol. Chem.* 281 1670–1679. 10.1074/jbc.M506776200 16278217

[B97] Nomura R. Naka S. Nemoto H. Inagaki S. Taniguchi K. Ooshima T. (2013). Potential involvement of collagen-binding proteins of *Streptococcus mutans* in infective endocarditis. *Oral Dis.* 19 387–393. 10.1111/odi.12016 22998492

[B98] Nomura R. Nakano K. Naka S. Nemoto H. Masuda K. Lapirattanakul J. (2012). Identification and characterization of a collagen-binding protein, Cbm, in *Streptococcus mutans*. *Mol. Oral Microbiol.* 27 308–323. 10.1111/j.2041-1014.2012.00649.x 22759315

[B99] Nomura R. Ogaya Y. Nakano K. (2016). Contribution of the collagen-binding proteins of *Streptococcus mutans* to bacterial colonization of inflamed dental pulp. *PLoS One* 11:e0159613. 10.1371/journal.pone.0159613 27442266 PMC4956251

[B100] Nylander Å Svensäter G. Senadheera D. B. Cvitkovitch D. G. Davies J. R. Persson K. (2013). Structural and functional analysis of the N-terminal domain of the *Streptococcus gordonii* adhesin Sgo0707. *PLoS One* 8:e63768. 10.1371/journal.pone.0063768 23691093 PMC3656908

[B101] Oehmcke S. Shannon O. Mörgelin M. Herwald H. (2010). Streptococcal M proteins and their role as virulence determinants. *Clin. Chim. Acta* 411 1172–1180. 10.1016/j.cca.2010.04.032 20452338

[B102] Patti J. M. Allen B. L. McGavin M. J. Höök M. (1994a). MSCRAMM-mediated adherence of microorganisms to host tissues. *Annu. Rev. Microbiol.* 48 585–617. 10.1146/annurev.mi.48.100194.003101 7826020

[B103] Patti J. M. Boles J. O. Hook M. (1993). Identification and biochemical characterization of the ligand binding domain of the collagen adhesin from *Staphylococcus aureus*. *Biochemistry* 32 11428–11435. 8218209 10.1021/bi00093a021

[B104] Patti J. M. Bremell T. Krajewska-Pietrasik D. Abdelnour A. Tarkowski A. Ryden C. (1994b). The *Staphylococcus aureus* collagen adhesin is a virulence determinant in experimental septic arthritis. *Infect. Immun.* 62 152–161. 8262622 10.1128/iai.62.1.152-161.1994 PMC186080

[B105] Patti J. M. House-Pompeo K. Boles J. O. Garza N. Gurusiddappa S. Hook M. (1995). Critical residues in the ligand-binding site of the *Staphylococcus aureus* collagen-binding adhesin (MSCRAMM). *J. Biol. Chem.* 270 12005–12011. 7744851 10.1074/jbc.270.20.12005

[B106] Paulsson M. Riesbeck K. (2018). How bacteria hack the matrix and dodge the bullets of immunity. *Eur. Respir. Rev.* 27:180018. 10.1183/16000617.0018-2018 29950304 PMC9488709

[B107] Phillips G. N. Jr. Flicker P. F. Cohen C. Manjula B. N. Fischetti V. A. (1981). Streptococcal M protein: alpha-helical coiled-coil structure and arrangement on the cell surface. *Proc. Natl. Acad. Sci. U.S.A.* 78 4689–4693. 10.1073/pnas.78.8.4689 7029524 PMC320228

[B108] Plumptre C. D. Ogunniyi A. D. Paton J. C. (2012). Polyhistidine triad proteins of pathogenic streptococci. *Trends Microbiol.* 20 485–493. 10.1016/j.tim.2012.06.004 22819099

[B109] PrabhuDas M. R. Baldwin C. L. Bollyky P. L. Bowdish D. M. E. Drickamer K. Febbraio M. (2017). A consensus definitive classification of scavenger receptors and their roles in health and disease. *J. Immunol.* 198 3775–3789. 10.4049/jimmunol.1700373 28483986 PMC5671342

[B110] Pyagay P. Heroult M. Wang Q. Lehnert W. Belden J. Liaw L. (2005). Collagen triple helix repeat containing 1, a novel secreted protein in injured and diseased arteries, inhibits collagen expression and promotes cell migration. *Circ. Res.* 96 261–268. 10.1161/01.RES.0000154262.07264.12 15618538

[B111] Reissmann S. Gillen C. M. Fulde M. Bergmann R. Nerlich A. Rajkumari R. (2012). Region specific and worldwide distribution of collagen-binding M proteins with PARF motifs among human pathogenic streptococcal isolates. *PLoS One* 7:e30122. 10.1371/journal.pone.0030122 22253902 PMC3256231

[B112] Rhem M. N. Lech E. M. Patti J. M. McDevitt D. Höök M. Jones D. B. (2000). The collagen-binding adhesin is a virulence factor in *Staphylococcus aureus* keratitis. *Infect. Immun.* 68 3776–3779. 10.1128/iai.68.6.3776-3779.2000 10816547 PMC97678

[B113] Ricard-Blum S. (2011). The collagen family. *Cold Spring Harb. Perspect. Biol.* 3:a004978. 10.1101/cshperspect.a004978 21421911 PMC3003457

[B114] Rich R. L. Kreikemeyer B. Owens R. T. LaBrenz S. Narayana S. V. Weinstock G. M. (1999). Ace is a collagen-binding MSCRAMM from *Enterococcus faecalis*. *J. Biol. Chem.* 274 26939–26945. 10480905 10.1074/jbc.274.38.26939

[B115] Ross C. L. Liang X. Liu Q. Murray B. E. Hook M. Ganesh V. K. (2012). Targeted protein engineering provides insights into binding mechanism and affinities of bacterial collagen adhesins. *J. Biol. Chem.* 287 34856–34865. 10.1074/jbc.M112.371054 22865854 PMC3464587

[B116] Sand J. M. B. Genovese F. Karsdal M. A. (2016). “Type IV collagen,” in *Biochemistry of Collagens, Laminins and Elastin*, Chap. 4, ed. Karsdal M. A. (Cambridge, MA: Academic Press), 31–41.

[B117] Sand J. M. B. Karsdal M. A. (2016). “Type XVI collagen,” in *Biochemistry of Collagens, Laminins and Elastin*, Chap. 16, ed. Karsdal M. A. (Cambridge, MA: Academic Press), 101–106.

[B118] Sato K. Yomogida K. Wada T. Yorihuzi T. Nishimune Y. Hosokawa N. (2002). Type XXVI collagen, a new member of the collagen family, is specifically expressed in the testis and ovary. *J. Biol. Chem.* 277 37678–37684. 10.1074/jbc.M205347200 12145293

[B119] Sato Y. Okamoto K. Kagami A. Yamamoto Y. Igarashi T. Kizaki H. (2004). *Streptococcus mutans* strains harboring collagen-binding adhesin. *J. Dent. Res.* 83 534–539. 10.1177/154405910408300705 15218042

[B120] Schönborn K. Willenborg S. Schulz J. N. Imhof T. Eming S. A. Quondamatteo F. (2020). Role of collagen XII in skin homeostasis and repair. *Matrix Biol.* 94 57–76. 10.1016/j.matbio.2020.08.002 32890632

[B121] Schönherr E. Hausser H. Beavan L. Kresse H. (1995). Decorin-type I collagen interaction. Presence of separate core protein-binding domains. *J. Biol. Chem.* 270 8877–8883. 10.1074/jbc.270.15.8877 7721795

[B122] Shen G. (2005). The role of type X collagen in facilitating and regulating endochondral ossification of articular cartilage. *Orthod Craniofac. Res.* 8 11–17. 10.1111/j.1601-6343.2004.00308.x 15667640

[B123] Shimoji Y. Ogawa Y. Osaki M. Kabeya H. Maruyama S. Mikami T. (2003). Adhesive surface proteins of *Erysipelothrix rhusiopathiae* bind to polystyrene, fibronectin, and type I and IV collagens. *J. Bacteriol.* 185 2739–2748. 12700253 10.1128/JB.185.9.2739-2748.2003 PMC154401

[B124] Shoulders M. D. Raines R. T. (2009). Collagen structure and stability. *Annu. Rev. Biochem.* 78 929–958. 10.1146/annurev.biochem.77.032207.120833 19344236 PMC2846778

[B125] Sillanpää J. Nallapareddy S. R. Qin X. Singh K. V. Muzny D. M. Kovar C. L. (2009). A collagen-binding adhesin, Acb, and ten other putative MSCRAMM and pilus family proteins of *Streptococcus gallolyticus* subsp. *gallolyticus* (*Streptococcus bovis* Group, biotype I). *J. Bacteriol.* 191 6643–6653. 10.1128/jb.00909-09 19717590 PMC2795296

[B126] Singh B. Fleury C. Jalalvand F. Riesbeck K. (2012). Human pathogens utilize host extracellular matrix proteins laminin and collagen for adhesion and invasion of the host. *FEMS Microbiol. Rev.* 36 1122–1180. 10.1111/j.1574-6976.2012.00340.x 22537156

[B127] Singh K. V. Nallapareddy S. R. Sillanpää J. Murray B. E. (2010). Importance of the collagen adhesin ace in pathogenesis and protection against *Enterococcus faecalis* experimental endocarditis. *PLoS Pathog.* 6:e1000716. 10.1371/journal.ppat.1000716 20072611 PMC2798748

[B128] Smeesters P. R. McMillan D. J. Sriprakash K. S. (2010). The streptococcal M protein: a highly versatile molecule. *Trends Microbiol.* 18 275–282. 10.1016/j.tim.2010.02.007 20347595

[B129] Speziale P. Raucci G. Visai L. Switalski L. M. Timpl R. Hook M. (1986). Binding of collagen to *Staphylococcus aureus* Cowan 1. *J. Bacteriol.* 167 77–81. 3722129 10.1128/jb.167.1.77-81.1986 PMC212843

[B130] Spivey K. A. Chung I. Banyard J. Adini I. Feldman H. A. Zetter B. R. (2012). A role for collagen XXIII in cancer cell adhesion, anchorage-independence and metastasis. *Oncogene* 31 2362–2372. 10.1038/onc.2011.406 21963851 PMC3968770

[B131] Stewart C. M. Buffalo C. Z. Valderrama J. A. Henningham A. Cole J. N. Nizet V. (2016). Coiled-coil destabilizing residues in the group A Streptococcus M1 protein are required for functional interaction. *Proc. Natl. Acad. Sci. U.S.A.* 113 9515–9520. 27512043 10.1073/pnas.1606160113 PMC5003295

[B132] Sun S. Karsdal M. A. (2016a). “Type VI collagen,” in *Biochemistry of Collagens, Laminins and Elastin*, Chap. 6, ed. Karsdal M. A. (Cambridge, MA: Academic Press), 49–55.

[B133] Sun S. Karsdal M. A. (2016b). “Type XVII collagen,” in *Biochemistry of Collagens, Laminins and Elastin*, Chap. 17, ed. Karsdal M. A. (Cambridge, MA: Academic Press), 107–111.

[B134] Sundaramoorthy M. Meiyappan M. Todd P. Hudson B. G. (2002). Crystal structure of NC1 domains. Structural basis for type IV collagen assembly in basement membranes. *J. Biol. Chem.* 277 31142–31153. 10.1074/jbc.M201740200 11970952

[B135] Taglialegna A. Matilla-Cuenca L. Dorado-Morales P. Navarro S. Ventura S. Garnett J. A. (2020). The biofilm-associated surface protein Esp of *Enterococcus faecalis* forms amyloid-like fibers. *NPJ Biofilms Microbiomes* 6:15. 10.1038/s41522-020-0125-2 32221298 PMC7101364

[B136] Tanaka T. Wakabayashi T. Oizumi H. Nishio S. Sato T. Harada A. (2014). CLAC-P/Collagen Type XXV is required for the intramuscular innervation of motoneurons during neuromuscular development. *J. Neurosci.* 34:1370. 10.1523/JNEUROSCI.2440-13.2014 24453327 PMC6705307

[B137] Tandon R. Sharma M. Chandrashekhar Y. Kotb M. Yacoub M. H. Narula J. (2013). Revisiting the pathogenesis of rheumatic fever and carditis. *Nat. Rev. Cardiol.* 10 171–177. 10.1038/nrcardio.2012.197 23319102

[B138] Theocharis A. D. Skandalis S. S. Gialeli C. Karamanos N. K. (2016). Extracellular matrix structure. *Adv. Drug Deliv. Rev.* 97 4–27. 10.1016/j.addr.2015.11.001 26562801

[B139] Tom Tang Y. Hu T. Arterburn M. Boyle B. Bright J. M. Palencia S. (2005). The complete complement of C1q-domain-containing proteins in Homo sapiens. *Genomics* 86 100–111. 10.1016/j.ygeno.2005.03.001 15953544

[B140] Ton Q. V. Leino D. Mowery S. A. Bredemeier N. O. Lafontant P. J. Lubert A. (2018). Collagen COL22A1 maintains vascular stability and mutations in COL22A1 are potentially associated with intracranial aneurysms. *Dis. Models Mech.* 11:dmm033654. 10.1242/dmm.033654 30541770 PMC6307901

[B141] Tonomura S. Ihara M. Kawano T. Tanaka T. Okuno Y. Saito S. (2016). Intracerebral hemorrhage and deep microbleeds associated with cnm-positive *Streptococcus mutans*; a hospital cohort study. *Sci. Rep.* 6:20074. 10.1038/srep20074 26847666 PMC4742798

[B142] Ton-That H. Schneewind O. (2004). Assembly of pili in Gram-positive bacteria. *Trends Microbiol.* 12 228–234. 10.1016/j.tim.2004.03.004 15120142

[B143] Vaca D. J. Thibau A. Schütz M. Kraiczy P. Happonen L. Malmström J. (2020). Interaction with the host: the role of fibronectin and extracellular matrix proteins in the adhesion of Gram-negative bacteria. *Med. Microbiol. Immunol.* 209 277–299. 10.1007/s00430-019-00644-3 31784893 PMC7248048

[B144] van Wieringen T. Kalamajski S. Lidén Å Bihan D. Guss B. Heinegård D. (2010). The streptococcal collagen-binding protein CNE specifically interferes with αVβ3-mediated cellular interactions with triple helical collagen. *J. Biol. Chem.* 285 35803–35813. 10.1074/jbc.M110.146001 20837478 PMC2975204

[B145] Veit G. Zwolanek D. Eckes B. Niland S. Käpylä J. Zweers M. C. (2011). Collagen XXIII, novel ligand for integrin alpha2beta1 in the epidermis. *J. Biol. Chem.* 286 27804–27813. 10.1074/jbc.m111.220046 21652699 PMC3149370

[B146] Waldemarsson J. Areschoug T. Lindahl G. Johnsson E. (2006). The streptococcal Blr and Slr proteins define a family of surface proteins with leucine-rich repeats: camouflaging by other surface structures. *J. Bacteriol.* 188 378–388. 10.1128/jb.188.2.378-388.2006 16385027 PMC1347292

[B147] Wang W. Olson D. Liang G. Franceschi R. T. Li C. Wang B. (2012). Collagen XXIV (Col24α1) promotes osteoblastic differentiation and mineralization through TGF-β/Smads signaling pathway. *Int. J. Biol. Sci.* 8 1310–1322. 10.7150/ijbs.5136 23139630 PMC3492790

[B148] Willumsen N. Karsdal M. A. (2016). “Type XX collagen,” in *Biochemistry of Collagens, Laminins and Elastin*, Chap. 20, ed. Karsdal M. A. (Cambridge, MA: Academic Press), 127–129.

[B149] Xu Y. Liang X. Chen Y. Koehler T. M. Hook M. (2004a). Identification and biochemical characterization of two novel collagen binding MSCRAMMs of *Bacillus anthracis*. *J. Biol. Chem.* 279 51760–51768. 10.1074/jbc.M406417200 15456768

[B150] Xu Y. Rivas J. M. Brown E. L. Liang X. Hook M. (2004b). Virulence potential of the staphylococcal adhesin CNA in experimental arthritis is determined by its affinity for collagen. *J. Infect. Dis.* 189 2323–2333. 10.1086/420851 15181582

[B151] Yu Z. An B. Ramshaw J. A. Brodsky B. (2014). Bacterial collagen-like proteins that form triple-helical structures. *J. Struct. Biol.* 186 451–461. 10.1016/j.jsb.2014.01.003 24434612 PMC4096566

[B152] Zainul Z. Heikkinen A. Koivisto H. Rautalahti I. Kallio M. Lin S. (2018). Collagen XIII is required for neuromuscular synapse regeneration and functional recovery after peripheral nerve injury. *J. Neurosci.* 38 4243–4258. 29626165 10.1523/JNEUROSCI.3119-17.2018 PMC6596032

[B153] Zani I. A. Stephen S. L. Mughal N. A. Russell D. Homer-Vanniasinkam S. Wheatcroft S. B. (2015). Scavenger receptor structure and function in health and disease. *Cells* 4 178–201. 10.3390/cells4020178 26010753 PMC4493455

[B154] Zhang Y.-Z. Ran L.-Y. Li C.-Y. Chen X.-L. (2015). Diversity, structures, and collagen-degrading mechanisms of bacterial collagenolytic proteases. *Appl. Environ. Microbiol.* 81 6098–6107. 10.1128/aem.00883-15 26150451 PMC4542243

[B155] Zong Y. Xu Y. Liang X. Keene D. R. Hook A. Gurusiddappa S. (2005). A ‘Collagen Hug’ model for *Staphylococcus aureus* CNA binding to collagen. *Embo J.* 24 4224–4236. 10.1038/sj.emboj.7600888 16362049 PMC1356329

